# Characterization of Extracellular Vesicles from Cell Suspension Cultures of *Coffea arabica* L.

**DOI:** 10.3390/plants15030439

**Published:** 2026-01-31

**Authors:** Azzurra Di Bonaventura, Dora Scarpin, Giacomo Trotta, Stefano Marchetti, Elisa Petrussa, Enrico Braidot, Luciano Navarini, Marco Zancani

**Affiliations:** 1 Department of Agricultural, Food, Environmental and Animal Sciences (DI4A), University of Udine, 33100 Udine, Italy; azzurra.dibonaventura@uniud.it (A.D.B.); dora.scarpin@uniud.it (D.S.); giacomo.trotta@uniud.it (G.T.); stefano.marchetti57@gmail.com (S.M.); elisa.petrussa@uniud.it (E.P.); 2 Illycaffè S.p.A., 34127 Trieste, Italy; luciano.navarini@illy.com

**Keywords:** cell suspension cultures, *Coffea arabica*, extracellular vesicles (EVs), morphometric traits, proteomics, unconventional protein secretion

## Abstract

A protocol was developed for the isolation and characterization of extracellular vesicles (EVs) from *Coffea arabica* cell suspension cultures (CSCs). The isolation method involved differential ultracentrifugation of the CSC filtrate, yielding two fractions: the pellet after 100,000×*g* for 36 min (100k×*g*) and the pellet obtained from the previous supernatant after 125,000 *g* for 6 h (125k×*g*). Both fractions were characterized by size, morphology, and proteomic profiles (ProteomeXchange identifier PXD071909). While no significant differences in average EV size were observed between the two fractions, proteomic analysis revealed distinct quantitative and compositional variations. The 100k×*g* fraction was enriched in proteins associated with cell periphery, plasma membrane, and extracellular region, whereas the 125k×*g* fraction predominantly contained proteins from the extracellular region. Proteomic marker analysis confirmed that both fractions contained protein EV markers, such as transmembrane and transport proteins, soluble EV-associated proteins, and proteins targeted to the extracellular environment or cell wall. Conversely, negligible contamination from non-EV-related proteins was detected. Furthermore, transmission electron microscopy (TEM) showed that the average size of the fractions was consistent with that reported for plant EVs. These findings demonstrate that the protocol utilized to isolate EVs from coffee CSC applies to the release of such vesicles without mechanical harsh grinding that leads to tissue/cell rupture and consequent contamination by other cell components. EVs obtained from coffee CSC represent a valuable and scalable platform, paving the way for the development of tools for biotechnological applications.

## 1. Introduction

The International Society for Extracellular Vesicles (ISEVs) recommended using the term “extracellular vesicles” (EVs) for “particles that are released from cells, are delimited by a lipid bilayer, and cannot replicate on their own (i.e., do not contain a functional nucleus)” [1]. This definition also encompasses EVs produced during cell migration or programmed cell death, called “migrasomes” and apoptotic bodies, respectively. EVs could also be distinguished on the basis of their dimensional features, being generally considered “small” when their diameter is <200 nm, even if there is no common consensus on upper and lower size cutoffs [1].

EVs are vesicles that have been extensively studied in mammals and found to be crucial for intercellular communication, as well as in the regulation of development and the progression of many diseases [2]. Plant-derived EVs nanovesicles possess many features similar to their mammalian counterparts [3]: their secretion has been associated with cell communication during growth and development [4], cell wall composition, and defense mechanisms against fungi, bacteria, and viruses [5].

In plants, EVs have been isolated by various methods, including (i) apoplastic washing, following vacuum infiltration and ultracentrifugation, from species such as *Arabidopsis thaliana*, *Nicotiana benthamiana*, and sunflower (*Helianthus annuus*) [6,7,8]; (ii) collection of plant exudates such as sap and root exudates [9,10,11]; (iii) isolation from cell suspension cultures (CSCs) [12,13,14]; (iv) mechanical extraction, including ginger (*Zingiber officinale*), carrot (*Daucus carota*) [15], broccoli (*Brassica oleracea* var. *italica*) [16], wheat (*Triticum aestivum*) [17], and dandelion (*Taraxacum officinale*) [18], or from citrus (*Citrus limon*) fruit [19], *Opuntia ficus indica* [20], grapefruit (*Citrus* × *paradisi*) [21], and watermelon (*Citrullus lanatus*) [22]. Nevertheless, it has been suggested that the generic term “plant EVs” should be associated with vesicular fractions obtained from spontaneously released EVs, as in the i–iii methodologies described above, while those obtained from destructive processes (or when their natural release cannot be clearly established) should be named “plant-derived nanovesicles” (PDNV) [12,23].

A further source of concern, beyond the plant source itself, is the highly variable protocols applied to isolate EVs. Differential ultracentrifugation (DUC) has been extensively utilized for plant materials since it is a reliable technique, although it is often adapted from protocols originally established for mammals. DUC is typically preceded by low-speed centrifugation (e.g., 500×*g*) to remove debris and organelles (e.g., [23]). Nevertheless, DUC speeds ranging from 40k×*g* to 100k×*g* can lead to heterogeneous EV populations, and longer or stronger DUC steps have been reported to promote the association of protein aggregates with the surface of EVs [24].

Much interest has arisen around the potential uses of plant EVs as biomedical therapeutics, offering many advantages over animal EVs: they are relatively easy and inexpensive to produce, are completely biocompatible, and readily absorbed by animal tissues without cross-species contamination. Additionally, plant EVs can encapsulate a diverse array of bioactive compounds, including proteins, lipids, small RNAs, and secondary metabolites, which might exert therapeutic effects on human health [12,25]. Supporting this, a recent study demonstrated that EV-like nanoparticles, obtained from hot roasted coffee (*Coffea arabica*), contain miRNAs capable of effectively inhibiting hepatocellular carcinoma proliferation [26]. This aligns with the long-recognized status of *Coffea* spp. as a rich source of bioactive compounds, with regular consumption of the most widely consumed beverage globally showing significant neuroprotective and liver-protective benefits [27]. Furthermore, biotechnology applications on *Coffea canephora* CSC have been developed to obtain extracts that are able to decrease oxidative stress, have anti-inflammatory properties, and provide protection from skin damage [28].

More promising, EVs from conditioned medium derived from cell cultures have also gained great interest for their large-scale and potentially more standardisable production in bioreactors [5,14]. Moreover, this biotechnology procedure of plant EV isolation avoids artificial cell destruction, ensuring the enrichment of more uniform populations of small extracellular vesicles.

In this work, we set up, for the first time to our knowledge, a reproducible and straightforward method for separating EVs from the conditioned medium of established CSC of *Coffea arabica* cv. Castillo [29] and characterized them based on their size, morphology, charge, and protein content. We tentatively propose this method as a reproducible protocol for EV isolation from plant CSCs, as it is easy, fast, and largely free of cellular contaminants. Furthermore, it paves the way for obtaining EVs from coffee CSC, which was selected because it is known to be a rich source of biologically active phytochemicals, including caffeine, phenolic compounds, and diterpenes that may exert beneficial effects on human health [28].

## 2. Results

### 2.1. Morphometric Characterization

**Figure 1:**
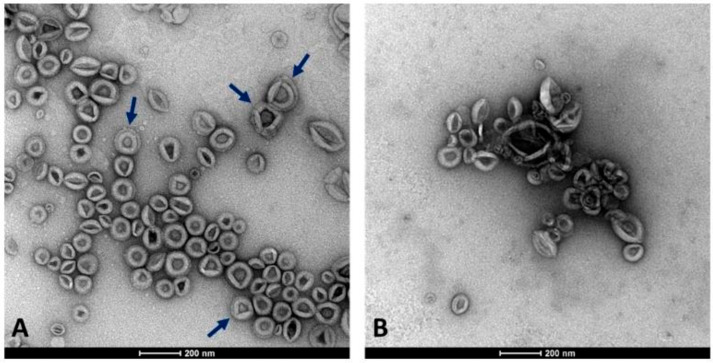
Appearance of the two EV fractions under the TEM microscope. (**A**) Fraction 100k×*g*. (**B**) Fraction 125k×*g*. Arrows highlight the presence in some vesicles with a double membrane layer. Scale bars represent 200 nm.

CSC represents an efficient and reliable system for producing useful compounds and serves as a valuable model for studying the release of EVs from plant cells. In this work, we isolated EVs by DUC from the conditioned medium collected from 2-week-old *C. arabica* CSC at the log-phase when they were characterized by high vitality and growth rate (Di Bonaventura et al., 2024 [29]). We aimed to separate two EV populations with different densities from the cell filtrate after removing cell debris, organelles, and microsomal fraction. For this purpose, the cleared cell filtrate was subjected to an initial high-speed ultracentrifugation at 100,000×*g* for 36 min, as a standard procedure usually applied for obtaining the denser microsomal vesicles. The supernatant obtained from the previous centrifugation underwent a second round of ultracentrifugation at higher centrifugal force and longer time (125,000×*g* for 6 h) to collect the nearly complete lighter fraction of membrane vesicles. Therefore, the two fractions were not separated by DUC at different speeds applied to the same supernatant, but by applying an additional step to the supernatant obtained after the first separation. As observed in TEM-derived images, EVs were unexpectedly obtained from both fractions, appearing as round-shaped vesicles with an apparent phospholipid membrane surrounding a dense interior and also revealing a relatively homogeneous size distribution (Figure 1).

The average vesicle diameters by TEM analysis fell within the size range typically associated with EVs (30–150 nm) (Table 1) [30]. Even though the vesicles were collected from separate fractions using different DUC protocols, no significant difference between the two populations was revealed by *t*-test analysis in their average diameters measured by TEM.

**Figure 2:**
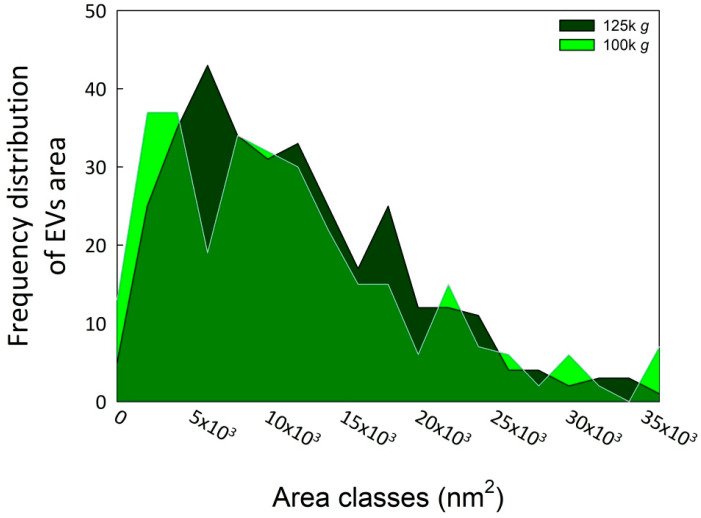
Area frequency distribution of the two EV fractions. The image analyses were performed using ImageJ Fiji software (Java version 1.8.0 322) [31]. The EV areas were calculated starting from at least 100 vesicles in each of three biological replicates for both fractions.

Subsequent morphometric analysis of TEM-derived images of EVs enabled the measurement of vesicle area, perimeter, and other morphometric parameters, including circularity, aspect ratio, roundness, and solidity, which collectively influence vesicle shape (results not shown). The two EV fractions were characterized by similar morphometric traits, with average vesicle areas (12,602 ± 7494 nm^2^ for 100k×*g* and 12,362 ± 8685 nm^2^ for 125k×*g*, *p* = 0.71) and perimeters (460.13 ± 151.26 nm for 100k×*g* and 455.22 ± 165.11 nm for 125k×*g*, *p* = 0.70) that were not significantly different between the two fractions. On the contrary, the aspect ratio (AR) ranged from 1.14 ± 0.12 in 100k×*g* to 1.22 ± 0.21 in 125k×*g* fraction, respectively. These results evidenced that both 125k×*g* and 100k×*g* EVs were elongated or elliptical vesicles (AR more than one), and that the 100k×*g* vesicles were significantly closer to a spherical shape compared to the 100k×*g* ones (*p* < 0.001). As expected, both fractions showed roundness values less than one, deviating from the perfect round shape, and also for this trait of the 100k×*g* fraction resulted in contain significantly more rounded-shaped EVs than the other samples (0.89 ± 0.08 vs. 0.84 ± 0.12, respectively, *p* < 0.001). Furthermore, the 100k×*g* fraction contained some EVs with double phospholipid bilayer (Figure 1, panel A, arrows). Despite the absence of a significant difference in their average area, the vesicle area distributions of the two samples were not fully overlapping (Figure 2), showing slight differences within the 5000 to 20,000 nm^2^ range.

### 2.2. Proteomic Analysis

**Figure 3:**
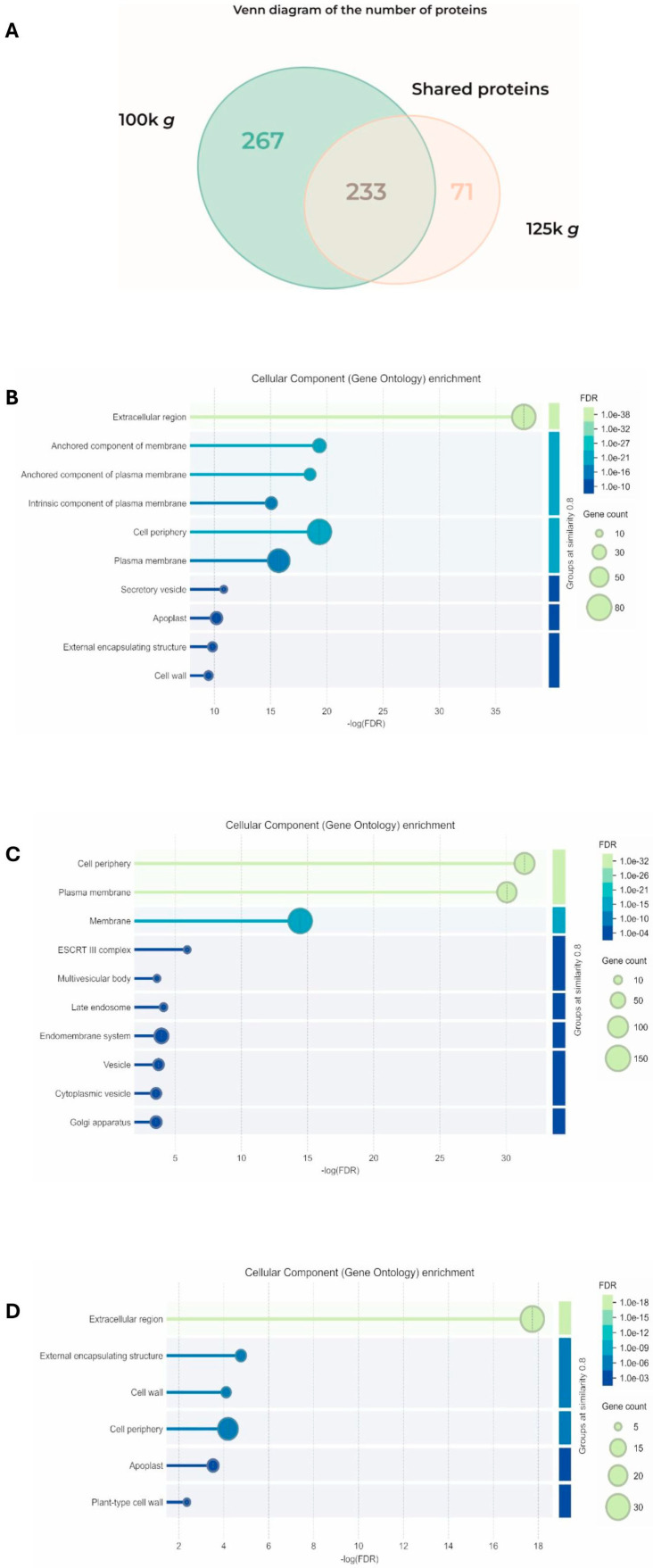
Analysis of the proteins detected in the 100k×*g* and 125k×*g* fractions. (**A**) Venn diagram showing the number of the shared proteins and those exclusive for each fraction. STRING analysis (**B**) of the 232 proteins shared by the two fractions (**C**) of the 267 proteins exclusive to the 100k×*g* fraction, and (**D**) of the 71 proteins exclusive to the 125k×*g* fraction, respectively, categorized based on cellular components. Gene ontology (GO) enrichment was performed using STRING software (Online version 12.0: https://string-db.org) [32], setting the similarity of groups at 0.8.

Proteomic analysis of the two fractions identified 499 and 303 proteins from 100k×*g* and 125k×*g*, respectively. The two populations were demonstrated to be separated, as demonstrated by Pearson correlation matrix (Figure S1). They shared 232 common proteins, while 267 proteins were exclusively detected in the 100k×*g* fraction and 71 were detected in the 100k×*g* fraction (Figure 3A).

These proteins were analyzed at both qualitative and quantitative levels using bioinformatic tools. A subset of protein markers proposed for EV characterization was also selected and further analyzed. A total of 37 uncharacterized proteins out of 499 (7.4%) and 15 out of 303 (5%) were present in the 100k×*g* fraction and in the 125k×*g* fraction, respectively. Regarding their localization, functional enrichment analysis of the 232 shared proteins using STRING [32] evidenced that they were primarily associated with extracellular compartments or to the plasma membrane (Figure 3B). Based on the FDR (False Discovery Rate) value, the most represented sequences shared by the two fractions were clustered as the Extracellular region (GO:0005576), containing 75 gene products (Supplementary Tables S1–S3). The 267 proteins exclusive to the 100k×*g* fraction were clustered as mainly associated with cell periphery and plasma membrane (Figure 3C), on the basis of functional enrichment analysis.

Based on the FDR parameter, the most represented sequences were clustered as Cell periphery (GO:0071944) and Plasma membrane (GO:0005886), containing 100 and 93 gene products, respectively (Supplementary Tables S4 and S5). Functional enrichment analysis of the 71 proteins exclusive to the 125k×*g* fraction revealed that they were primarily associated with apoplast/extracellular compartments (Figure 3D). According to the FDR parameter, the most represented sequences were clustered as the Extracellular region (GO:0005576), containing 30 gene products (Supplementary Tables S6 and S7).

**Figure 4:**
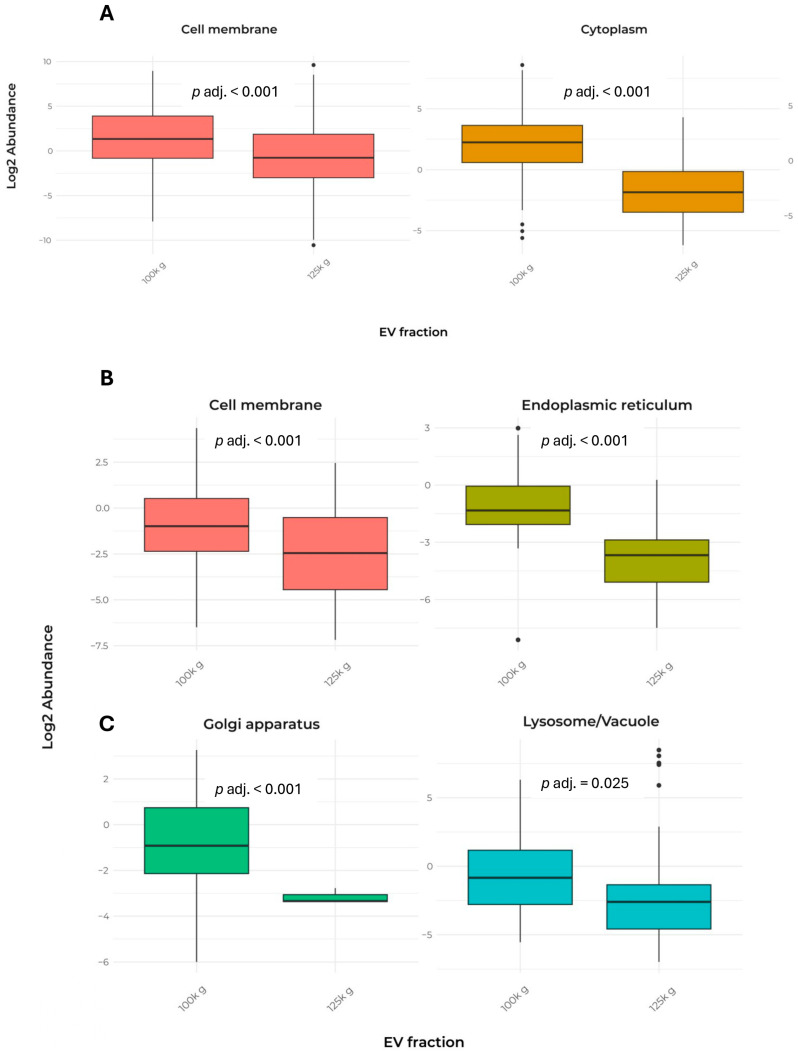
Comparison of EV protein abundance in cell compartments in the two fractions. The data report only the localizations that showed significant differences between the two fractions. (**A**) Box plot of the abundance of proteins shared by the 100k×*g* and 125k×*g* fractions. (**B**,**C**) Box plot of the abundance of proteins exclusive to the 100k×*g* or 125k×*g* fraction, respectively. Box plots show only the groups that exhibited a significant difference (*p* adj. ≤ 0.05). Protein localization was predicted using DeepLoc-2.1, a web interface tool, which estimates the most probable cell localization on the basis of the highest probable value. Proteins were divided into nine categories (i.e., cell membrane, cytoplasm, endoplasmic reticulum, extracellular, Golgi apparatus, vacuole/lysosome, mitochondrion, nucleus, plastid).

In addition, Table 2 presents the cellular localization and the number of detected peptides used for the identification of the 232 proteins shared between the two fractions. The data indicated that both fractions exhibited a largely similar pattern, except for cell membrane and cytoplasm proteins (Figure 4A).

Remarkably, only a few proteins from mitochondria, nucleus, plastid, and Golgi apparatus were detected, indicating a low contamination by membranes released when cell disruption occurs. In contrast, the analysis of exclusive proteins from each fraction revealed significant differences in cellular localization, particularly in lysosome/vacuole, cell membrane, endoplasmic reticulum, and Golgi apparatus (Table 3 and Figure 4B,C).

Molecular characterization of the two EVs fractions was then carried out in more detail, considering only those proteins so far proposed as markers for plant-derived nanometric-sized vesicles. Initially the identified protein sequences were classified based on the established guidelines for animal EVs, then refined with regard to what was recently proposed for EVs’ characterization in the plant kingdom (see Materials and Methods). After applying a qualitative approach, Table 4 and Supplementary Table S8 show that the 42 proteins in Group 1 (membrane or transport proteins) were largely represented by the Ser/Thr-protein kinases found mostly in the 100k×*g* fraction. 

**Table 4:** Distribution of the plant EV marker protein accessions present in *C. arabica* proteome, in the subset of shared proteins, and exclusive to either the 100k×*g* or 125k×*g* fractions. The proteins are organized into five functional categories, with the percentages for each marker group shown in brackets.

Class	Gene ID (*Coffea arabica*) (570 Total Accessions)	Shared Accessions (232 Accessions)	Accessions Exclusive to the 100k×*g* Fraction (267 Accessions)	Accessions Exclusive to the 125k×*g* Fraction (71 Accessions)
**Group 1: transmembrane or GPI-anchored proteins**	**42 acc. (7.4%)**	**11 acc. (4.7%)**	**23 acc. (8.6%)**	**8 acc. (11.3%)**
**TET8**				
Tetraspanins membrane proteins [23,33,34,35,36]	2 (0.4%)	2 (0.9%)	0 (0%)	0 (0%)
**PEN1**				
Penetration1 intraluminal protein Syntaxins [34,36]	5 (0.9%)	3 (1.3%)	2 (0.7%)	0 (0%)
**Ser/Thr-protein kinases**				
classified together with G-type lectin S-receptor-like and involved in stress [9,12]	35 (6.1%)	6 (2.6%)	21 (7.9%)	8 (11.3%)
**Group 2: cytosolic proteins recovered in EVs**	**48 acc. (8.4%)**	**7 acc. (3.0%)**	**34 acc. (12.7%)**	**7 acc. (9.9%)**
**HSP70**				
Heat shock protein 70 [12]				1 (1.4%)
**ANXA**				
Annexin [12,33,37]	1 (0.2%)		1 (0.4%)	
**PATL3**				
Patellin or Patellin-like intraluminal proteins [34,36,37,38]	1 (0.2%)		1 (0.4%)	
**Actins**				
Actins and Actin-like proteins [35,39]	4 (0.7%)	1 (0.4%)	2 (0.7%)	1 (1.4%)
**GTPase Rab-type**				
Rab-like GTPase marker of MVB (multi-vesicular bodies) [5,23,33]	29 (5.1%)	4 (1.7%)	23 (8.6%)	2 (2.8%)
**RIN4**				
RPM1-Interacting Protein 4 [5,37,40]	2 (0.4%)		2 (0.7%)	
**Endosomal Sorting Complex Required for Transport (ESCRT)-related proteins**				
Involved in MVB and intraluminal vesicles formation, ESCRT involves about 20 proteins (e.g., ESCRT-0, -I, -II, and -III, AAA ATPase Vps4 complex) [5,41]	5 (0.9%)		5 (1.9%)	
**Ser carboxypeptidase**				
Serine carboxypeptidases could act in plant as acyl transferase [35]	5 (0.9%)	2 (0.9%)		3 (4.2%)
**Group 3: Major components of non-EV co-isolated structures**	**None**	**None**	**None**	**None**
**Group 4: Transmembrane, lipid-bound and soluble proteins associated with other intracellular compartments than PM/endosomes**	**36 acc. (6.3%)**	**6 acc. (2.6%)**	**23 acc. (8.6%)**	**7 acc. (9.9%)**
**Mitochondrion**	3 (0.5%)	1 (0.4%)	2 (0.7%)	0 (0%)
**Plastid**	17 (3.0%)	2 (0.9%)	12 (4.5%)	3 (4.2%)
**Nucleus**	13 (2.3%)	3 (1.3%)	7 (2.6%)	3 (4.2%)
**Peroxisomes**	3 (0.5%)	0 (0%)	2 (0.7%)	1 (1.4%)
**Group 5: Cell Wall Remodeling/Degrading Enzymes (CWREs/CWDEs) and Pathogenesis-Related (PR) proteins**	**66 acc. (11.6%)**	**44 (19.0%)**	**4 acc. (1.5%)**	**18 acc. (25.4%)**
**Glucosidases**				
glycosyl hydrolase family 3 proteins (α or β-1,3-Glucosidase, generally carbohydrate hydrolytic enzymes)[12,23,35]	22 (3.9%)	17 (7.3%)	0 (0%)	5 (7.0%)
**Pectinesterases**				
Cell wall biogenesis/degradation Pectinesterase/pectinesterase inhibitor 51 [12,35,37]	8 (1.4%)	5 (2.2%)	0 (0%)	3 (4.2%)
**Chitinases**				
Class V chitinases and endochitinases [9,35]	17 (3.0%)	12 (5.2%)	1 (0.4%)	4 (5.7%)
**Peroxidases**				
[35,42]	11 (1.9%)	7 (3.0%)	2 (0.7%)	2 (2.8%)
**BBE-like proteins**				
Berberine-bridge enzyme-like 8, -18 and -21, related to oxidative reactions [12]	8 (1.4%)	3 (1.3%)	1 (0.4%)	4 (5.7%)
**Total accession number of groups 1, 2 and 5**	**156/570 (27.4%)**	**62/232 (26.7%)**	**61/267 (22.8%)**	**33/71 (46.5%)**

The remaining proteins in this group were subdivided almost evenly between the 125k×*g* and shared fractions. Likewise, the 48 soluble cytosolic proteins in Group 2 were largely associated with the 100k×*g* fraction, particularly the Rab-type GTPase proteins. The absence of exomers and supermeres (nucleotide and protein polymers without a lipid coating comprised in Group 3 [43,44]) cannot be excluded, since the TEM images are not sufficiently resolved to evidence them. In addition, no lipoproteins, which are considered EV contaminants in animals [45], were detected in both fractions.

Another qualitative criterion is provided by Group 4, which includes membrane-associated proteins from intracellular compartments other than plasma membrane and/or endosomes. In this case, the low number of accessions in this group (36 out of 570, or 6.3%) suggests good enrichment of EVs in the two fractions. Although the 100k×*g* fraction contains the highest number of these “negative” markers (23 accessions) in terms of absolute value, they represent just a low percentage (8.6%) of its total proteins, confirming a limited contamination from other endo-membranes. Among the different marker classes, proteins from Group 5 (CWREs/CWDEs and PR proteins) were the most prevalent. The majority of these cell wall-shaping proteins were found in the shared subset and in the 125k×*g* fraction. Specifically, in the latter fraction, the detected proteins belonging to Group 5 accounted for 25%.

**Figure 5:**
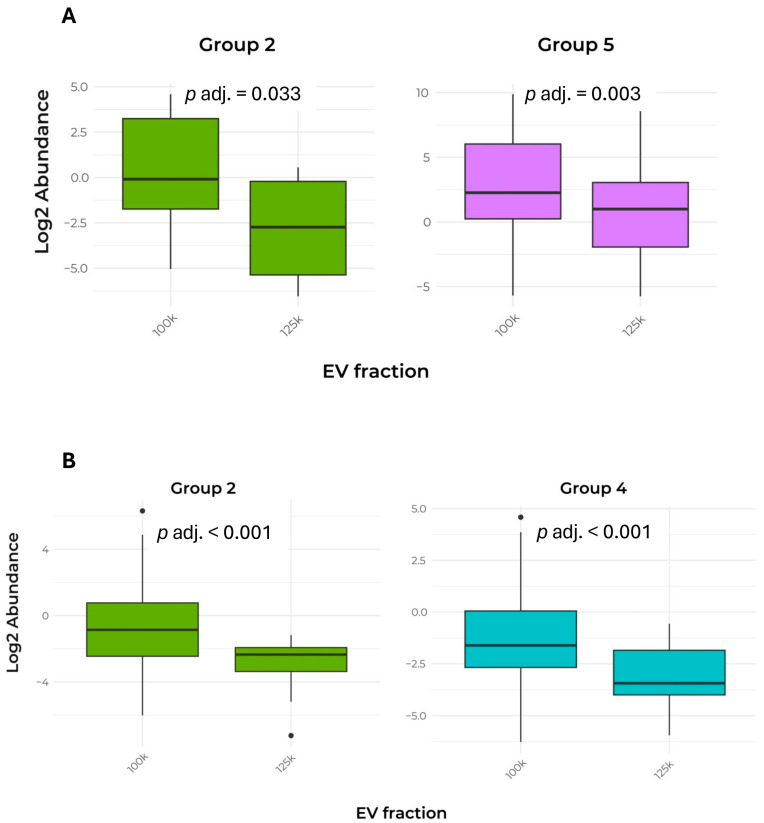
Comparison of abundance in EV marker proteins between the two fractions. (**A**) Box plot of the abundance of Groups 2 and 5 protein markers shared by the 100k×*g* and 125k×*g* fractions. (**B**) Box plot of the abundance of Groups 2 and 4 protein markers exclusive to either the 100k×*g* or 125k×*g* fraction. Box plots show only the groups that exhibited a significant difference (*p* adj. ≤ 0.05). The groups were defined as follows: Group 2, cytosolic proteins recovered in EVs; Group 4, transmembrane, lipid-bound, and soluble proteins associated with other intracellular compartments than plasma membrane/endosomes; Group 5, CWREs/CWDEs and PR proteins.

Overall, the molecular marker analysis provided encouraging results regarding the presence of EVs obtained from coffee CSC. Across all fractions, approximately one-fourth of the detected accessions corresponded to EV markers (Groups 1, 2 and 5) [1], with this proportion increasing to nearly 50% in the 125k×*g* fraction (Table 4). An overview of the EV marker proteins shared by both fractions is provided by quantitative approach in Table 5 and Figure 5A.

The relative abundance (Log_2_ transformation) of these marker proteins was analyzed using parametric or non-parametric tests to identify significant differences between the two fractions. Notably, the protein abundance in Groups 2 (cytosolic proteins inside the vesicles) and 5 (CWREs/CWDEs and PR proteins) was significantly higher in the 100k×*g* fraction when compared to the 125k×*g* fraction. The pattern of marker protein distribution, considering the Log_2_ abundance of gene products exclusively present in each fraction, is described in Table 6 and Figure 5B.

The parametric *t*-test shows a significant difference between the two fractions, with the 100k×*g* fraction containing a higher abundance of protein markers of Groups 2 and 4. This quantitative approach again confirmed that Group 2 EV markers represent the key factors in discriminating the two fractions. Remarkably, in the case of Group 2, these results are consistent with the increase observed in the shared proteins (see Table 5 and Figure 5A).

## 3. Discussion

Extracellular vesicles from plant sources have recently attracted significant attention. Nevertheless, the widespread isolation and use of plant EVs as carriers of metabolites and biologically active molecules are still limited by the absence of standardized protocols that ensure compositional uniformity. In addition, the use of non-destructive separation methods that can enrich crude extracts in EVs with less contamination from proteins, nucleotides, or other subcellular components resulting from harsh extraction conditions is also recommended [11]. EVs are predominantly isolated through the physical disruption of plant tissues, such as fruits and stems, or by juicing sample processing [46]. While these techniques yield a large quantity of EVs, they raise questions regarding the actual origin of the membranes and potential contamination introduced during the disruption process. Alternatively, less invasive methods have been employed to collect EVs, such as vacuum infiltration extraction [8] or collection from root exudates [9].

In line with the most commonly used methods for enriching fractions obtained from plant cell cultures [24], which closely resemble the isolation procedures employed for fluids from mammalian cell cultures, we established a protocol for EV enrichment from the culture medium filtrate of *Coffea arabica* cv. Castillo. Specifically, we adopted the DUC technique, which is widely used in both plant and animal systems due to its simplicity and reproducibility [37]. This method enables the removal of contaminants, such as cell debris and organelles, through preliminary low-speed centrifugation steps.

Furthermore, the use of axenic cell cultures minimized the risk of bacterial and fungal contamination, while initial filtration and preliminary centrifugation steps removed coarse particles from the supernatants, followed by DUC. Unexpectedly, the first pellet, obtained after centrifugation at 100,000×*g* for 36 min of the supernatant derived from the preliminary centrifugation steps, was not only largely free of major contaminants—such as transmembrane, lipid-bound, and soluble proteins associated with intracellular compartments other than the plasma membrane (PM) or endosomes—but also yielded a pellet (100k×*g* fraction) containing EVs detectable by DLS and TEM analysis. Further centrifugation of the previous supernatant at 125,000×*g* (125k×*g* fraction) did not significantly alter the characteristics or morphology of the precipitated vesicles (Figure 1 and Figure 2).

TEM analyses (Table 1) indicate that EVs derived from coffee CSC fall within the size range commonly reported for plant-derived EVs whose diameters are below 500 nm, typically between ~50 and 200 nm. Furthermore, the two fractions exhibited very similar size distributions (Figure 2), despite the 125k×*g* fraction being obtained from the supernatant of the 100k×*g* fraction. Nevertheless, Figure S1 shows that the two fractions exhibited good reproducibility among biological replicates, but belonged to distinct populations with respect to their proteomic profiles. A hypothetical explanation of this behavior relies on the uncomplete pelleting of EVs after the first DUC at 100k×*g* for 36 min, which might represent a sedimentation threshold that is insufficient to obtain the whole EV population. Indeed, when the previous supernatant was subjected to DUC at 125k×*g* for 6 h, the pellet was further enriched. Additionally, these findings indicate the co-presence of proteins transported within vesicles that differ in composition and cargo loading [40] and that can be separated by DUC [47].

A posteriori, the choice to perform an initial ultracentrifugation step to precipitate microsomal vesicles, appeared to be an unnecessary precaution, given the minimal contamination observed in the 100k×*g* fraction. Nevertheless, it served to confirm the high degree of EV enrichment achievable even with a relatively low DUC speed and also demonstrated the feasibility of obtaining EV-enriched fractions from CSC with minimal contamination, even without employing highly stringent sedimentation protocols such as step-gradient centrifugation, size-exclusion chromatography, immuno-affinity purification, or anion-exchange chromatography [30,36,48]. We suggest that vesicle extrusion in coffee CSC could be facilitated by the presence of a plastic cell wall, characteristic of young and actively dividing plant cells, which is neither highly thickened nor lignified. Moreover, this cell wall may act as a sieve, physically restricting the passage of larger vesicles, and thereby influencing their size distribution, as reflected by the skewness in the vesicle size distribution (Figure 2).

Despite the similarity in EV morphology between the two fractions, they exhibit distinct protein profiles which, although partially overlapping (46.5% shared with the 100k×*g* fraction and 74.4% with the 125k×*g* fraction), also reveal specific and unique protein content exclusive to each fraction (Figure 3A). The protein distribution between the two fractions revealed a substantial shared portion (232 peptides), confirming significant overlap between the high-density 100k×*g* and low-density 125k×*g* fractions. Based on STRING analysis, a distinct qualitative pattern of proteins was evident between the two fractions under study. These proteins were primarily associated with the extracellular region and, to a lesser extent, with membrane environments, such as the PM and cell periphery (Figure 3B and Supplementary Table S1). These latter compartments were also found to be enriched in the 100k×*g* fraction (Figure 3C and Supplementary Tables S3–S5), suggesting a differential EV delivery compared to the 125k×*g* fraction, where the extracellular domain showed the lowest FDR index (Figure 3D and Supplementary Tables S6 and S7). The physiological relevance of these observations may relate to the need to extrude low-density EVs for long-distance transport and to target them toward the cell wall, as supported by the presence of proteins involved in biotic and abiotic stress responses, as well as in cell wall remodeling (Supplementary Tables S2 and S7).

Quantitative analysis of peptides confirmed that shared proteins were targeted to specific cellular compartments to different extents when the 100k×*g* and 125k×*g* fractions were compared (Figure 4 and Table 2). Statistical analysis of proteomics of the two vesicle populations showed that proteins delivered by the 100k×*g* fraction were more likely to localize at the PM or in regions immediately peripheral to the protoplast, with significantly higher probability compared to the 125k×*g* fraction. In contrast, the 125k×*g* fraction predominantly contained proteins destined for the outermost apoplastic zone, with significant enrichment in proteins involved in cell wall and middle lamella remodeling, as well as in responses to biotic stresses [9,37,49]. A similar protein distribution was observed in cell cultures of Norway spruce (*Picea abies*), which release EVs containing enzymes involved in lignin synthesis—such as laccases, peroxidases, β-glucosidases, and putative dirigent proteins—as well as enzymes recruited for cell wall remodeling, including glycosyl hydrolases, transglucosylases/hydrolases, and expansins [50].

According to the EV classification criteria recently proposed for plant-derived extracellular vesicles [11,34,35,40,51,52], a number of characteristic markers have been identified and grouped on the basis on their functional properties. In particular, the MISEV guidelines [1,23,53] recommend that EV characterization be validated through the identification of distinct groups of markers: (1) membrane proteins, or those potentially involved in transport, indicative of the presence of lipid bilayer structures (e.g., tetraspanins); (2) soluble cytosolic proteins that interact with membranes via receptor recognition or anchoring processes (e.g., Rab proteins such as ARA6, and annexins); (3) proteins from non-EV structures that may co-purify with EVs (e.g., non-vesicular extracellular nanoparticles); (4) cytosolic proteins from intracellular compartments other than the PM or endosomal system. A further class, namely secreted proteins recovered with EVs, has been proposed as the fifth group by MISEV [1]. Even if further experiments with protease protection assays are necessary to confirm the enclosure and/or the association of these marker proteins in EVs, in the case of plants, we suggest that this characterizing group might include CWREs/CWDEs [49,54,55], evidenced in sunflower [42], *C. arabica*, thale cress (*Arabidopsis thaliana*) [56], Norway spruce [57], kiwifruit (*Actinidia deliciosa*) [58], and PR proteins [59,60,61] found in sunflower [42], olive (*Olea europea*) [62], thale cress [37], *Brassica oleracea* [60], and potato (*Solanum tuberosum*) [35], as specific plant EV markers. We propose the adoption of such further marker class as a helpful contribution in the complex characterization of plant EVs. In fact, this group not only underlines the well-known involvement of EVs in the pathogen/host interaction in plant disease but also explains the presence of hydrolytic enzymes in EVs as functional factors that are essential for overcoming the cell wall barrier.

In accordance with these criteria, another characterization was performed by analyzing proteins defined as EV markers. The analysis focused on three groups of detected marker proteins: those shared between fractions, and those exclusive to either the 100k×*g* or 125k×*g* fractions. Both qualitative and quantitative approaches were applied, with the quantitative analysis following the same experimental procedures previously described for protein localization.

The qualitative distribution of proteins within the groups proposed as EV markers [5,9,12,23,33,34,35,36,37,39,40,41,42,53,56,60] is detailed in Table 4 and Supplementary Table S8. The EV markers collectively identified in the two fractions accounted for a substantial portion of the entire coffee EV proteome, representing 27.4%, 26.7%, and 22.8% of the total proteins, the shared proteins, and those exclusive to the 100k×*g* fraction, respectively. Remarkably, the percentage of EV marker proteins identified in the 125k×*g* fraction approaches 50%. These results confirm that the supernatant of coffee CSC is a valuable source of EVs, even when a simple separation method such as DUC was used. Nevertheless, these vesicle-enriched fractions need to be further investigated to assess the degree of purity by purification approaches including size-exclusion chromatography, density gradient flotation, and protease protection assays [1].

We propose that these EVs are produced through a physiological process in which cells, during their development and growth in liquid media, spontaneously release extracellular vesicles into the surrounding environment, or alternatively, in response to biotic and abiotic stress, as suggested by Maricchiolo et al. [63] and Rabouille [64]. These authors have described an Unconventional Protein Secretion (UPS) pathway that bypasses the Golgi and trans-Golgi network route and is conserved across mammals, yeast, and plants. This secretory mechanism plays roles in cell wall reorganization, plant defense, and interspecific cross-talk with environmental factors. EVs derived from cultures of plant cell suspensions, calli, hairy roots, and pollen have been recently reviewed by Ambrosone et al. [5]. Although studies are still limited, they highlight the potential for obtaining EV production that is standardizable, scalable, contaminant-free, and bio-sustainable. Notably, consistent with our findings, EVs have been isolated from CSC of tobacco (*Nicotiana tabacum* L.) [12,65], Korean ginseng (*Panax ginseng* C.A. Meyer) [66], blue carpet (*Craterostigma plantagineum* Hochst.) [12], and kalimeris (*Aster yomena*) [67]. Our results align closely with these studies, particularly regarding vesicle size and morphology. Indeed, the mechanism by which EVs traverse the barrier formed by the cell wall remains poorly understood. Interestingly, the explanation provided by Brown et al. [68] for fungi and bacteria, which also have cell walls, could also be valid in the case of plant cell cultures. Such a hypothesis involves three different coexisting pathways, involving turgor pressure, loosening of the cell wall by hydrolytic enzymes, and the presence of protein channels, confirmed by the detection of actin and tubulin in EV preparations.

Regarding the possible origin of coffee EVs, based on the proteomic analysis of proteins in the two fractions—which contained proteins mainly derived from the cytoplasm, membrane-associated/cell periphery, and apoplast/extracellular environment—we suggest that these vesicles represent a mixed population. They could originate either from multi-vesicular body (MVB) formation via the Type III pathway (organelle-based translocation and extracellular vesicles), as proposed by Maricchiolo et al. [63]; from plasma membrane budding, similar to what has been observed in yeast and animal cells [69] and in plants [5,12,70]; or from the Exocyst-Positive Organelle (EXPO) secretion pathway [63]. In the latter case, cytosolic proteins would be secreted via an UPS pathway through an as-yet-unknown mechanism. MVBs, on the other hand, are likely generated from the endoplasmic reticulum (ER) by bypassing the Golgi apparatus, and are subsequently transported to the plasma membrane, where they fuse and release their contents into the apoplast. Notably, the exclusive protein profile of the 100k×*g* fraction showed a higher abundance of cytosolic and ER proteins—and unexpectedly, also Golgi proteins (Table 2 and Table 3; Figure 4)—along with a lower amount of extracellular proteins compared to the 125k×*g* fraction. This observation sustains the hypothesis that in the 125k×*g* fraction, the primary mechanism of EV formation may be plasma membrane budding, with soluble proteins potentially recruited for cell wall remodeling (belonging to Group 5). A similar protein pattern was found in CSC of *Picea abies* that release EVs, containing enzymes involved in lignin synthesis (such as laccases, peroxidases, β-glucosidases, putative dirigent proteins), or recruited in cell wall modeling (such as glycosyl hydrolases, transglucosylases/hydrolases, and expansins [50]). Conversely, in the 100k×*g* fraction Rab-like GTPases, markers of MVB included in Group 2 are largely represented (Table 4), suggesting their involvement in delivering proteins related to signaling and cell communication.

Concerning the quantitative aspects of protein EV markers, the two fractions were similar (Figure 5; Table 5 and Table 6), suggesting that both fractions are representative of plant EVs, although they exhibited a distinct distribution of specific proteins. In particular, the analysis of the abundance of shared and exclusive protein markers of Group 2 shows that the 100k×*g* fraction was enriched in EVs containing cytosolic proteins. In addition, protein markers of Group 4 in the exclusive portion (Table 6 and Figure 5B) and those of Group 5 in the shared portion were again significantly increased in the 100k×*g* fraction, confirming its higher density. Through observing the early precipitation by DUC of most proteins, we demonstrated that long duration (e.g., 6 h) and high gravity force (e.g., 125k×*g*) would not be necessary to obtain an adequate EV harvest from coffee CSC in terms of quality and quantity.

## 4. Materials and Methods

### 4.1. Chemicals

2,4,5-trichlorophenoxyacetic acid (2,4,5-T), kinetin (KT), and myo-inositol were purchased from Duchefa Biochemie (Haarlem, The Netherlands); other chemicals were purchased from Sigma-Aldrich Chemie (Steinheim, Germany).

### 4.2. Cell Suspension Cultures

CSCs were obtained as described in Di Bonaventura et al. [29]. Briefly, CSCs from *Coffea arabica*, cv. Castillo were started by transferring 2 g FW of 21-day-old calluses into 250 mL Erlenmeyer baffled flasks (Corning, New York, NY, USA) with vent caps, each containing 50 mL MS medium supplemented with 1 mg/L KT and 1 mg/L 2,4,5-T, 30 g/L sucrose and 1 mg/L myo-inositol. CSCs were placed on a rotary shaker (110 rpm, 26 ± 1 °C, in the dark) (MaxQ600, Thermo Fisher Scientific, Cincinnati, OH, USA) and sub-cultured every 10–12 days by transferring 10 mL of suspension to 40 mL of fresh medium.

### 4.3. Extracellular Vesicle Isolation

EVs were isolated from CSC by Differential Ultra-Centrifugation (DUC) following the protocol described by Braidot et al. [71] and modified as below. The culture medium was separated from 2-week-old CSC by filtration (Schott Duran Mainz, Germany sintered glass filter, 16–40 µm pore size) and preliminarily centrifuged at 2500×*g* for 6 min (SS34 Sorvall rotor) and 12,000×*g* for 12 min (SS34 Sorvall rotor, Osterode, Germany). The pellet was discarded, and the supernatant was subjected to DUC at 100,000×*g* for 36 min (Type 70 Ti Beckman Coulter rotor, Miami, FL, USA) and the pellet was collected (named 100k×*g*). The previous supernatant was again subjected to DUC at 125,000×*g* for 6 h (Type 70 Ti Beckman Coulter rotor) and the pellet was collected (125k×*g*). Both the 100k×*g* and 125k×*g* pellets were resuspended in phosphate-buffered saline (PBS) of pH 7.4, with 25 mM trehalose and stored at −80 °C.

### 4.4. Determination of EV Size and Shape

Transmission Electron Microscopy (TEM) images were obtained by placing a drop of 100k×*g* or 125k×*g* suspensions (approximately 25 µL) onto a 400-mesh holey film grid. After staining with 2% (*w*/*v*) uranyl acetate for 2 min, the samples were observed using a Tecnai G2 (Thermo Fisher Scientific FEI Deutschland GmbH, Dreieich, Germany)) transmission electron microscope operating at 120 kV. Images were captured with a Veleta digital camera (Olympus Soft Imaging System). The images were then processed by the *Labkit* plugin [72] of *ImageJ Fiji* software (Java version 1.8.0 322) to measure the following morphometric parameters: area (nm^2^), diameter (nm), roundness (R, 4 × [area]/{π × [major axis]^2^}), aspect ratio (AR, [major axis]/[minor axis]), circularity (C, 4π × [area]/[perimeter]^2^), and solidity (S, [area]/[convex area]) [73].

### 4.5. Proteomic Sample Preparation

The 100k×*g* or 125k×*g* samples (three biological replicates for each fraction) were resuspended in RIPA buffer (25 mM Tris-HCl, pH 7.6, 150 mM NaCl, 1% NP-40, 1% sodium deoxycholate, 0.1% SDS) containing protease inhibitors and incubated at 4 °C for 30 min. Samples were then sonicated (30 s on/30 s off cycle, amplitude 35, for 10 min) using a Q700 Sonicator (Qsonica, Newtown, CT, USA) and clarified by centrifugation at 14,000 *g* for 15 min. The supernatants were collected and subjected to TCA-deoxycholate precipitation for 2 h at 4 °C. Protein pellets were washed with ice-cold 80% acetone and resuspended in 2% (*w*/*v*) SDS, 50 mM HEPES, with pH 8.0. Subsequently, samples were measured for protein content using a BCA assay. For each sample, proteins (30 µg) were reduced with 10 mM DTT for 45 min at 56 °C and alkylated in 22.5 mM iodoacetamide for 30 min in the dark. The reaction was quenched to a final concentration of 10 mM DTT. Sample digestion, protein, and peptide clean-up were then performed according to the single-pot solid-phase-enhanced sample preparation (SP3) method [74,75]. Briefly, 6 μL of the bead mix (1:1 hydrophilic and hydrophobic beads at 50 µg/µL, GE Life Sciences) was added to the samples. Afterward, acetonitrile (ACN) was added to a final concentration of 70% (*v*/*v*), and samples were mixed at room temperature for 20 min. After the beads had settled, the supernatant was removed, and the beads were washed twice with 70% (*v*/*v*) ethanol (EtOH) and once with 100% ACN. Beads were resuspended in 50 μL of 100 mM ammonium bicarbonate, 1 mM CaCl_2_, and digested overnight with trypsin (1:20 trypsin: protein ratio) at 37 °C under mixing (1000 rpm). The following day, peptides were collected by incubation on a magnetic rack and subjected to peptide clean-up. A total of 9 μL of fresh carboxyl magnetic bead mix (50 µg/µL, GE Life Sciences) was mixed with each sample, and ACN was added such that the final ACN concentration was 95% (*v*/*v*). The mixtures were incubated for 10 min at RT with shaking. The beads were then washed twice with ACN, and peptides were eluted with 50 μL of 2% (*v*/*v*) ACN in water by mixing the samples on a rotor for 5 min. Before LC-MS/MS analysis, the peptides mixture was centrifuged at 20,000 *g* for 5 min to remove bead carryover, and the supernatant was collected and acidified to a final concentration of 0.1% (*v*/*v*) formic acid.

### 4.6. Liquid Chromatography Tandem Mass Spectrometry (LC-MS/MS) Analysis

Digested samples were separated using an Easy-nLC 1200 system (Thermo Fisher Scientific, San Jose, CA, USA) and loaded onto a reversed-phase column (Acclaim PepMap RSLC C18 column 2 µm particle size, 100 Å pore size, id 75 µm, Thermo Fisher Scientific, San Jose, CA, USA); they were heated at 40 °C, with a two-component mobile phase system of 0.1% (*v*/*v*) formic acid in water (buffer A) and 0.1% (*v*/*v*) formic acid in ACN (buffer B). Peptides were eluted using a gradient of 5% to 25% over 55 min, followed by 25% to 40% over 10 min, and 40% to 98% over 10 min; the peptides were kept at 98% over 10 min, with a flow rate of 200 nL/min. Samples were injected in an Orbitrap Fusion Tribrid mass spectrometer (Thermo Fisher Scientific, San Jose, CA, USA) and data were acquired in a data-dependent mode (2200 V). Full scans were performed at 120,000 FWHM resolving power (at 200 *m*/*z*) and an AGC target of 1 × 10^6^. A mass range of 350–1100 m/z was surveyed for precursors, with first mass set at 140 m/z for fragments. Each full scan was followed by a set of MS/MS scans (HCD, collision energy of 30%) over 3 s cycle time at 150 ms maximum injection time (ion trap) and AGC target of 5 × 10^3^. The ion transfer tube temperature was set at 200 °C. Dynamic exclusion was enabled and set to 40 s, with a mass tolerance of 5 ppm.

### 4.7. Proteomic Data Analysis

Peptide searches were performed in the Proteome Discoverer 2.2 software (Thermo Fisher Scientific) against the *Coffea arabica* database (UniProt UP000515148, August 2024), including a list of major known contaminants. Proteins were identified using MASCOT search engine with a mass tolerance of 10 ppm for precursors and 0.6 Da for produced ions. Trypsin was chosen as the enzyme with five missed cleavages, and static modification of carbamidomethyl (C) with variable modification of oxidation (M) and acetyl (protein N-term) were incorporated in the search. False Discovery Rate (FDR), which estimates the expected proportion of false positives, was filtered with *p* < 0.01 at PSM, peptide, and protein level. Results were filtered to exclude potential contaminants. A minimum of three peptides was considered to identify a protein. Peak intensities were transformed into Log_2_ space. Data were normalized by the average of its abundance within each sample to account for variation in sampling volumes [76]. The mass spectrometry proteomics data have been deposited to the ProteomeXchange Consortium via the PRIDE [77] partner repository with the dataset identifier PXD071909 and 10.6019/PXD071909. The reproducibility of protein content among different biological replicates (three for each fraction) was assessed using Pearson correlation matrix. Gene ontology (GO) enrichment was performed using the STRING software [32], setting the similarity of groups at 0.8.

### 4.8. Protein Localization Analysis

Protein localization was predicted using DeepLoc-2.1, a web interface tool. Preliminarily, the list with all the proteins was collected and converted into FASTA format through the usage of a Python script (version 3.12.4; Van Rossum and Drake 2009 [78]). After the processing with DeepLoc-2.1, a dataset with the probability of protein localizations divided into nine categories (i.e., cell membrane, cytoplasm, endoplasmic reticulum, extracellular, Golgi apparatus, vacuole/lysosome, mitochondrion, nucleus, plastid) was obtained. The most probable localization was selected on the basis of the highest probable value [79]. The dataset was then integrated with the Log_2_ abundance value for each protein, categorized by treatment (i.e., 100k×*g* and 125k×*g*) and biological replicate (*n* = 3). Log_2_ transformation is used in proteomic analyses to normalize data distribution, stabilize variance, and simplify fold-change interpretation, making the data more suitable for statistical analysis and visualization [76]. The same procedure was applied to the subset of proteins that have been suggested as markers of plant EVs. According to these criteria, these proteins were organized into five groups to characterize the extracellular nature of the vesicles, after identification by the proteomic data analysis performed on both fractions. The five groups were defined as follows, according to the literature [5,9,12,23,33,34,35,36,39,40,41,42,56]: (i) Group 1: transmembrane or GPI-anchored proteins; (ii) Group 2: cytosolic proteins recovered in EVs; (iii) Group 3: major components of non-EV co-isolated structures; (iv) Group 4: transmembrane, lipid-bound and soluble proteins associated with other intracellular compartments than plasma membrane/endosomes; (v) Group 5: CWREs/CWDEs and PR proteins.

### 4.9. Statistical Analysis

Statistical analyses to test the differences between the two isolation methods were performed using the R software (Version 4.4.1; R Core Team 2024 [80]). For morphometric analysis, *t*-test was applied to verify if the two EV populations of the 100k×*g* and 125k×*g* showed differences about the morphometric parameters reported above. Regarding the proteomic analyses, the normality of the abundance value distribution was first tested (i.e., Shapiro–Wilk’s test). If the distribution was not normal, a further check was performed using QQ-plot for normality. To analyze the differences between abundance, localization, and treatments, a *t*-test was performed. Data that failed to follow normality were then analyzed using non-parametric analyses (i.e., Wilcoxon test). The model tested the differences in terms of Log_2_ abundance in relation to the treatment (i.e., Log_2_ abundance ~ Treatment). The “summary” function was used to extract the *p*-adjusted value. The *p*-value was considered significant for values < 0.05. The “ggplot2” R package was used to visualize the significant box plots [81]. This approach was performed at the levels of cell localization and EV markers.

## 5. Conclusions

In the present study, we developed a straightforward and effective method using DUC to isolate two distinct fractions enriched in EVs that were spontaneously released from coffee CSC. Our results indicate that these EVs are produced physiologically, rather than arising from cell homogenization or degenerative processes, such as cell death or necrosis. This conclusion is supported by the minimal contamination of proteins associated with various organelles and membrane systems, including the nucleus, mitochondria, vacuoles, and plastids. The characterization of the EV-enriched fractions has been performed mainly by proteomic analysis, providing an initial description of the components associated with these vesicles.

Although the EV yield is relatively low due to the limited starting material, the coffee CSCs we previously established [29] provide a scalable platform for increasing liquid culture volume via bioreactor cultivation. This approach could facilitate the production of larger quantities of EVs, potentially enriched with proteins, short chains of nucleic acids, and secondary metabolites possessing bio-pharmacological properties. Although additional studies are needed to fully characterize the content of coffee CSC-derived EVs and improved purification techniques may further refine their composition, they could provide a valuable tool in biotechnology. CSC-derived EVs might represent delivery vehicles for valuable coffee secondary metabolites or carriers loaded with nutraceutical or pharmaceutical compounds, opening promising perspectives for applications in biopharming, medical treatments, and therapies.

## Figures and Tables

**Table 1:** Average vesicle diameter measured by TEM analysis, performed using Fiji software (*win-64* version) [31]. Data were obtained from the analysis of at least 100 vesicles in each of three biological replicates for both fractions.

Fraction		
100k *g* Avg Diameter ± SD (nm)	125k *g* Avg Diameter ± SD (nm)	*t*-Test
120.90 ± 37.84	117.81 ± 43.22	n.s.

n.s., not significant.

**Table 2:** Quantitative analysis of relative abundance (Log_2_ transformation) and cellular localization of the proteins shared by the 100k×*g* and 125k×*g* fractions. The number of detected peptides was calculated on the basis of their presence in three biological replicates. Bold figures indicate a significant difference in relative abundance between the two fractions for a specific cellular localization (*p* adj. ≤ 0.05). Protein localization was predicted using DeepLoc-2.1, a web interface tool, which estimates the most probable cell localization on the basis of the highest probable value. Proteins were divided into nine categories (i.e., cell membrane, cytoplasm, endoplasmic reticulum, extracellular, Golgi apparatus, vacuole/lysosome, mitochondrion, nucleus, plastid). Statistical analysis was performed using parametric (*t*-test) or non-parametric (Wilcoxon) test depending on the normal distribution of the data.

Test	Localization	N. Peptides (%)		Statistics	df	*p*	*p* Adj.
		100k×*g*	125k×*g*				
Non-parametric	Extracellular	234	249	29,846		0.642	1
(Wilcoxon test)	Lysosome/Vacuole	111	112	6677		0.339	1
Parametric	Cell membrane	198	207	6.18	403	<0.001	**<0.001**
(*t*-test)	Cytoplasm	53	52	7.17	97.9	<0.001	**<0.001**
	ER	16	17	0.95	28.9	<0.001	1
	Golgi apparatus	9	9	1.7	15.2	<0.001	0.99
	Mitochondrion	3	3	0.871	2.5	<0.001	1
	Nucleus	9	9	2.28	15.3	<0.001	0.333
	Plastid	5	5	−0.784	5.52	<0.001	1

**Table 3:** Quantitative analysis of relative abundance (Log_2_ transformation) and cellular localization of the proteins exclusively presents in either the 100k×*g* or 125k×*g* fraction. The number of detected peptides was calculated based on their presence across the three biological replicates. Bold figures indicate a significant difference in relative abundance between the two fractions for a specific cellular localization (*p* adj. ≤ 0.05). Protein localization was predicted using DeepLoc-2.1, a web interface tool, which estimates the most probable cell localization on the basis of the highest probable value. Proteins were divided into nine categories (i.e., cell membrane, cytoplasm, endoplasmic reticulum, extracellular, Golgi apparatus, vacuole/lysosome, mitochondrion, nucleus, plastid).

Test	Localization	N. Peptides (%)		Statistics	df	*p*	*p* Adj.
		100k×*g*	125k×*g*	Wilcoxon Test			
Non- parametric	Cytoplasm	234	10	1708		0.030	0.269
	Lysosome/ Vacuole	70	40	1883		0.003	**0.025**
				*t*-test			
Parametric	Extracellular	55	63	1.56	116	0.122	1
	Cell membr.	255	37	3.10	46.3	0.003	**0.029**
	ER	23	20	4.35	40.9	<0.001	**<0.001**
	Golgi	43	3	5.54	23.6	<0.001	**<0.001**
	Nucleus	20	9	1.44	26.8	0.162	1
	Plastid	31	6	3.20	8.12	0.012	0.112

**Table 5:** Quantitative analysis of the relative abundance (Log_2_ transformation) of marker proteins shared by the 100k×*g* and 125k×*g* fractions. Peptide counts were determined from their detection across the three biological replicates. Bold figures indicate a significant difference in relative abundance between the two fractions for a specific marker group (*p* adj. ≤ 0.05). Group 3 was not analyzed because no accession was present in the shared fraction. Statistical analysis was performed using parametric (*t*-test) or non-parametric (Wilcoxon) test depending on the normal distribution of the data. The groups were defined as follows: Group 1, transmembrane or GPI-anchored proteins; Group 2, cytosolic proteins recovered in EVs; Group 4, transmembrane, lipid-bound and soluble proteins associated with other intracellular compartments than plasma membrane/endosomes; Group 5, CWREs/CWDEs and PR proteins.

Test	Marker Group	N. Peptides		Statistics	df	*p*	*p* Adj.
		100k×*g*	125k×*g*				
Non-parametric (Wilcoxon test)	Group 1	34	28	617		0.047	0.188
	Group 5	149	84	7925		0.0007	**0.003**
Parametric							
(*t*-test)	Group 2	16	18	−2.98	31.7	0.008	**0.033**
	Group 4	14	14	−1.94	24.2	0.064	0.257

**Table 6:** Quantitative analysis of the relative abundance (Log_2_ transformation) of marker proteins exclusive to either the 100k×*g* or 125k×*g* fraction. Peptide counts were determined from their detection across the three biological replicates. Bold figures indicate a significant difference in relative abundance between the two fractions for a specific marker group (*p* adj. ≤ 0.05). Statistical analysis was performed using parametric (*t*-test) or non-parametric (Wilcoxon) test depending on the normal distribution of the data. The groups were defined as follows: Group 1, transmembrane or GPI-anchored proteins; Group 2, cytosolic proteins recovered in EVs; Group 4, transmembrane, lipid-bound, and soluble proteins associated with other intracellular compartments than plasma membrane/endosomes; Group 5, CWREs/CWDEs and PR proteins.

Test	Marker Group	N. Peptides		Statistics	df	*p*	*p* Adj.
		100k×*g*	125k×*g*				
Non-parametric	Group 5	9	50	255		0.534	1
(Wilcoxon test)							
Parametric	Group 1	63	20	1.26	27.9	0.218	0.872
(*t*-test)	Group 2	98	18	4.90	33.4	<0.001	**<0.001**
	Group 4	65	20	3.96	47.8	<0.001	**<0.001**

## Data Availability

Data are contained within the article and in the Supplementary Materials.

## Supplementary Materials

The following supporting information can be downloaded at https://www.mdpi.com/article/10.3390/plants15030439/s1; Figure S1. Pearson correlation matrix comparing the proteomics profile of 100k×*g* and 125k×*g* samples. Pairwise correlations were calculated using Pearson’s correlation coefficient. Variables can be considered correlated when |r| > 0.75. Asterisks indicate the statistical significance of correlations; Table S1: GO analysis and list of proteins shared by the two fractions; Table S2: List of the 75 proteins shared by the two fractions, clustered as the Extracellular region (GO:0005576); Table S3: GO analysis and list of proteins exclusive to the 100k×*g* fraction; Table S4: List of the 100 proteins exclusive to the 100k×*g* fraction, clustered as Cell periphery (GO:0071944); Table S5: List of the 93 proteins exclusive to the 100k×*g* fraction, clustered as Plasma membrane (GO:0005886); Table S6: GO analysis and list of proteins exclusive to the 125k×*g* fraction; Table S7: List of the 30 proteins exclusive to 125k×*g* fraction, clustered in the Extracellular region (GO:0005576); Table S8: List of plant EV marker protein accessions in the subset of shared and exclusive proteins from either the 100k×*g* or 125k×*g* fractions. Markers are listed by accession number and organized into five functional categories. N.d., not detected. Gene IDs in brackets represent synonyms of the preceding entry, according to UniprotKB database. Shared accessions and gene loci are highlighted in bold. The symbol ✓ indicates the presence of shared proteins in either the 100k×*g* or 125k×*g* fraction.

## Abbreviations

The following abbreviations are used in this manuscript:

2,4-D	2,4-dichlorophenoxyacetic acid
CSC	Cell suspension cultures
CWREs/CWDEs	Cell wall remodeling/degrading enzymes
DLS	Dynamic light scattering
DUC	Differential ultra-centrifugation
ESCRT	Endosomal sorting complex required for transport
EVs	Extracellular vesicles
EXPO	Exocyst-positive organelle
FDR	False discovery rate
GO	Gene ontology
KT	Kinetin
LC-MS/MS	Liquid chromatography tandem mass spectrometry
MVB	Multi-vesicular bodies
PR	Pathogenesis-related
TEM	Transmission electron microscopy
UPS	Unconventional Protein Secretion
