# Supplementary material for: Characterization of Extracellular Vesicles from Cell Suspension Cultures of Coffea arabica L
Source: Plants (Basel). 2026 Jan 31;15(3):439. doi: 10.3390/plants15030439 (PMC12899826; doi:10.3390/plants15030439)
Supplement: Supplementary file 1 [file plants-15-00439-s001.zip › plants-4028364-supplementary.pdf]

Characterization of extracellular vesicles from cell suspension cultures of *Coffea arabica* L.

Azzurra Di Bonaventura[1], Dora Scarpin[1], Giacomo Trotta[1], Stefano Marchetti[1], Elisa Petrussa[1], Enrico Braidot[1*], Luciano Navarini[2], Marco Zancani[1]

SUPPLEMENTARY MATERIAL

Figure S1. Pearson correlation matrix comparing the proteomics profile of 100k×*g* and 125k×*g* samples. Pairwise correlations were calculated using Pearson's correlation coefficient. Variables can be considered correlated when |r| > 0.75. Asterisks indicate the statistical significance of correlations.

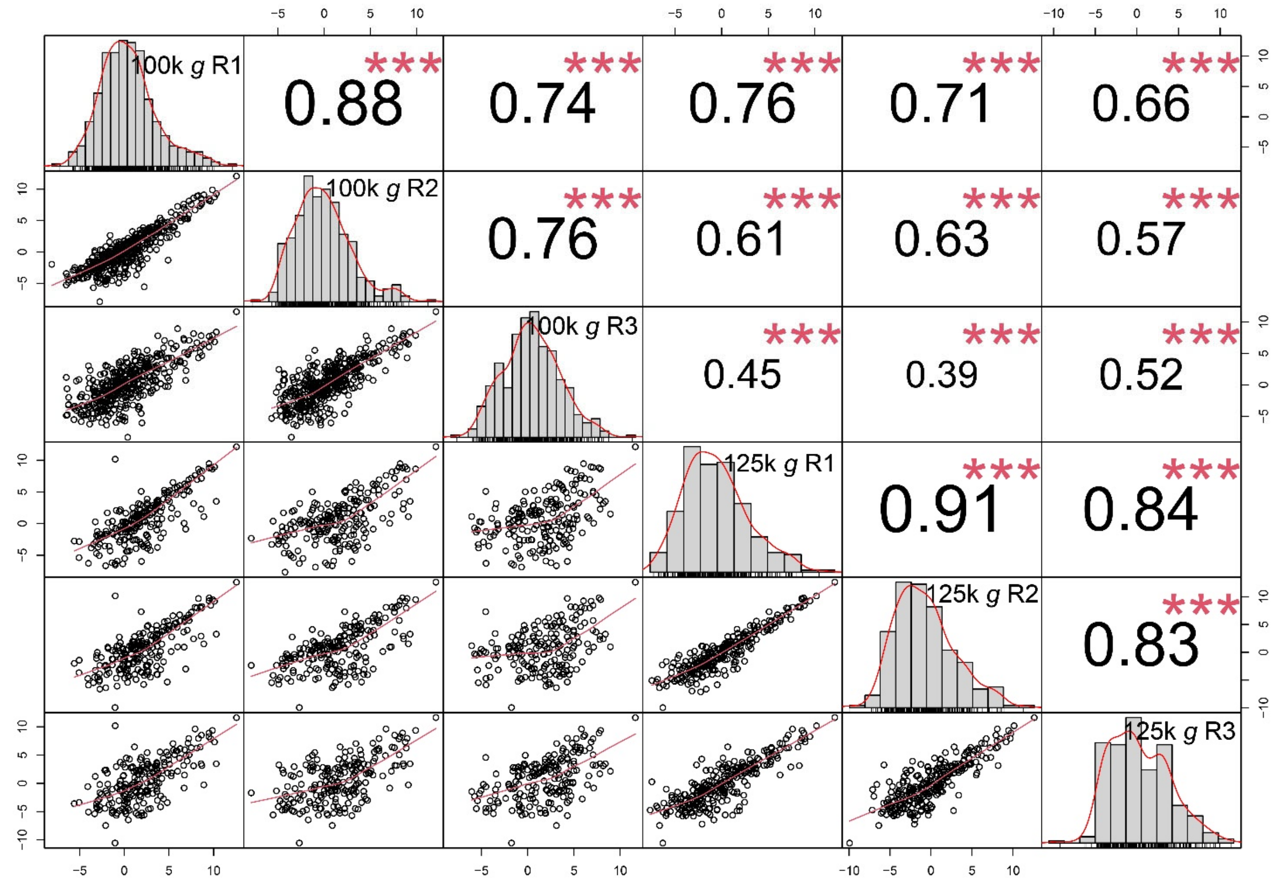

Table S1: GO analysis and list of proteins shared by the two fractions.

	Cellular Component (Gene Ontology)				
**GO-term**	**Description**	**Count in network**	**Strength**	**Signal**	**False discovery rate**
GO:0005576	Extracellular region	75 of 2491	0.84	2.84	$3.13e^{-38}$
GO:0031225	Anchored component of membrane	27 of 420	1.17	3.28	$4.58e^{-20}$
GO:0071944	Cell periphery	75 of 4892	0.55	1.45	$4.58e^{-20}$
GO:0046658	Anchored component of plasma membrane	22 of 246	1.31	3.58	$3.13e^{-19}$
GO:0005886	Plasma membrane	65 of 4387	0.53	1.34	$1.91e^{-16}$
GO:0031226	Intrinsic component of plasma membrane	23 of 424	1.10	2.64	$8.60e^{-16}$
GO:0099503	Secretory vesicle	13 of 134	1.35	2.44	$1.44e^{-11}$
GO:0048046	Apoplast	24 of 825	0.82	1.62	$6.48e^{-11}$
GO:0030312	External encapsulating structure	17 of 366	1.03	1.89	$1.46e^{-10}$
GO:0005618	Cell wall	16 of 330	1.05	1.87	$3.28e^{-10}$

Table S2. : List of the 75 proteins shared by the two fractions, clustered as Extracellular region (GO:0005576).

UniProt		Protein identification
**1.**	A0A6P6SE81	Peroxidase
**2.**	A0A6P6SI03	Beta-glucosidase
**3.**	A0A6P6SI29	Cysteine proteinase 3-like
**4.**	A0A6P6SJ83	Peroxidase
**5.**	A0A6P6SJT4	Xyloglucan endotransglucosylase/hydrolase
**6.**	A0A6P6SKC8	Class V chitinase-like
**7.**	A0A6P6SKY1	Xyloglucan endotransglucosylase/hydrolase
**8.**	A0A6P6SL89	Beta-galactosidase
**9.**	A0A6P6SM06	Pectinesterase
**10.**	A0A6P6SMR6	Beta-glucosidase
**11.**	A0A6P6SNE2	Class V chitinase-like
**12.**	A0A6P6SR39	Class V chitinase-like
**13.**	A0A6P6SSD2	EG45-like domain containing protein
**14.**	A0A6P6SV85	Peroxidase
**15.**	A0A6P6SVG7	Xyloglucan endotransglucosylase/hydrolase
**16.**	A0A6P6SYG0	Class V chitinase-like
**17.**	A0A6P6SZV1	Xyloglucan endotransglucosylase/hydrolase
**18.**	A0A6P6T2W0	Non-reducing end alpha-L-arabinofuranosidase
**19.**	A0A6P6T4J5	Peroxidase
**20.**	A0A6P6TBD6	Subtilisin-like protease SBT1.7
**21.**	A0A6P6THJ8	Peroxidase
**22.**	A0A6P6TJC1	Subtilisin-like protease SBT1.7
**23.**	A0A6P6TJY0	STS14 protein
**24.**	A0A6P6TL51	Basic endochitinase-like
**25.**	A0A6P6TR46	Heparanase-like protein 3
**26.**	A0A6P6TW52	Serine carboxypeptidase-like 2
**27.**	A0A6P6U6B2	Cysteine proteinase inhibitor
**28.**	A0A6P6U6L5	Beta-galactosidase
**29.**	A0A6P6U6Q2	Berberine bridge enzyme-like 21
**30.**	A0A6P6U6X2	Berberine bridge enzyme-like 21 isoform X1
**31.**	A0A6P6UBP3	Alpha-glucosidase
**32.**	A0A6P6UCF7	Expansin-like A2
**33.**	A0A6P6UEU9	Glucan endo-1,3-beta-glucosidase 1-like
**34.**	A0A6P6UHF7	Berberine bridge enzyme-like 21 isoform X1
**35.**	A0A6P6UJ34	Pathogenesis-related protein 5-like
**36.**	A0A6P6UK81	Kunitz trypsin inhibitor 2-like
**37.**	A0A6P6ULD1	Xyloglucan endotransglucosylase hydrolase protein 22-like
**38.**	A0A6P6UNQ4	Low-temperature-induced cysteine proteinase-like
**39.**	A0A6P6UPD9	Cysteine-rich repeat secretory protein 55-like
**40.**	A0A6P6USL3	Cysteine-rich repeat secretory protein 55-like

**41.**	A0A6P6UUQ8	Pathogenesis-related protein 5-like
**42.**	A0A6P6UWG9	Serine carboxypeptidase-like 7
**43.**	A0A6P6UXX0	Low-temperature-induced cysteine proteinase-like
**44.**	A0A6P6UY93	Non-specific lipid transfer protein GPI-anchored 1-like
**45.**	A0A6P6V0U3	chitinase
**46.**	A0A6P6V2V7	Basic 7S globulin-like
**47.**	A0A6P6V570	Basic 7S globulin-like
**48.**	A0A6P6VBR3	Acidic endochitinase-like
**49.**	A0A6P6VC73	Alpha-xylosidase 1-like
**50.**	A0A6P6VCB2	Non-specific lipid transfer protein GPI-anchored 1-like
**51.**	A0A6P6VJA7	GDSL esterase/lipase At5g14450
**52.**	A0A6P6VSQ7	Pathogenesis-related protein 1A-like
**53.**	A0A6P6VT21	GDSL esterase/lipase At1g29670-like
**54.**	A0A6P6VTC4	Peroxidase
**55.**	A0A6P6VWQ5	Carboxypeptidase
**56.**	A0A6P6VWU7	Carboxypeptidase
**57.**	A0A6P6VWW4	Beta-xylosidase/alpha-L-arabinofuranosidase 2-like
**58.**	A0A6P6VXM4	Pectinesterase
**59.**	A0A6P6VXM9	beta-glucosidase
**60.**	A0A6P6VYA3	Germin-like protein
**61.**	A0A6P6W961	Pectinesterase
**62.**	A0A6P6W9A2	Peroxidase
**63.**	A0A6P6WBD9	Cysteine proteinase RD21A-like
**64.**	A0A6P6WCX1	Beta-xylosidase/alpha-L-arabinofuranosidase 2-like
**65.**	A0A6P6WEP0	Carboxypeptidase
**66.**	A0A6P6WFL0	Carboxypeptidase
**67.**	A0A6P6WFR6	Pathogenesis-related protein 1A-like
**68.**	A0A6P6WMP4	Purple acid phosphatase
**69.**	A0A6P6WPW5	Cell wall hydroxyproline-rich glycoprotein
**70.**	A0A6P6WUQ5	Purple acid phosphatase
**71.**	A0A6P6WUT2	Xyloglucan endotransglucosylase/hydrolase
**72.**	A0A6P6XI18	Peroxidase
**73.**	A0A6P6XIA7	EP1-like glycoprotein 2
**74.**	A0A6P6XIZ3	Alpha-galactosidase
**75.**	A0A6P6XKP1	Beta-galactosidase

16

Table S3: GO analysis and list of proteins exclusive to the 100k×*g* fraction.

	Cellular Component (Gene Ontology)				
**GO-term**	**Description**	**Count in network**	**Strength**	**Signal**	**False Discovery Rate**
GO:0071944	Cell periphery	100 of 4892	0.61	1.87	$6.86e^{-33}$
GO:0005886	Plasma membrane	93 of 4387	0.63	1.91	$1.47e^{-31}$
GO:0016020	Membrane	140 of 14593	0.28	0.88	$1.23e^{-15}$
GO:0000815	ESCRT III complex	5 of 9	2.05	1.6	$1.24e^{-06}$
GO:0005770	Late endosome	8 of 127	1.1	0.99	$7.59e^{-05}$
GO:0012505	Endomembrane system	45 of 4148	0.34	0.59	0.00011
GO:0031982	Vesicle	22 of 1341	0.52	0.67	0.00018
GO:0005771	Multivesicular body	5 of 38	1.42	0.95	0.00024
GO:0031410	Cytoplasmic vesicle	21 of 1294	0.51	0.65	0.00028
GO:0005794	Golgi apparatus	26 of 1859	0.45	0.61	0.00028

Table S4: List of the 100 proteins exclusive to the 100k×*g* fraction, clustered as Cell periphery (GO:0071944).

	UniProt	Protein identification
**1.**	A0A6P6S9C1	Protein NOI4-like
**2.**	A0A6P6S9G2	Protein NOI4-like
**3.**	A0A6P6SA02	Protein NDR1-like
**4.**	A0A6P6SHH5	FT-interacting protein 7-like
**5.**	A0A6P6SIP3	Serine/threonine-protein kinase isoform X1
**6.**	A0A6P6SK98	Calcium-dependent protein kinase 8-like
**7.**	A0A6P6SQQ6	Vacuolar protein sorting-associated protein 32 homolog 2
**8.**	A0A6P6SR90	Auxin transporter-like protein 2
**9.**	A0A6P6SRR2	Probable serine/threonine-protein kinase PBL17
**10.**	A0A6P6SRV0	Sugar transport protein 7-like
**11.**	A0A6P6SUL9	FT-interacting protein 7-like
**12.**	A0A6P6SVT4	Calcium-dependent protein kinase 8-like
**13.**	A0A6P6SYV1	Heavy metal-associated isoprenylated plant protein 6
**14.**	A0A6P6SZX5	Probable serine/threonine-protein kinase PBL8 isoform X1
**15.**	A0A6P6T2R3	VAMP-like protein YKT61
**16.**	A0A6P6T3C0	Auxin transporter-like protein 2
**17.**	A0A6P6T443	Temperature-induced lipocalin-1-like
**18.**	A0A6P6T638	Probable receptor-like protein kinase At2g23200
**19.**	A0A6P6T779	Nucleobase-ascorbate transporter 11-like
**20.**	A0A6P6T7R8	PI-PLC X domain-containing protein At5g67130-like
**21.**	A0A6P6T9D6	Uncharacterized protein LOC113698779
**22.**	A0A6P6T9G7	Non-specific serine/threonine threonine-protein kinase
**23.**	A0A6P6TA99	Protein MID1-COMPLEMENTING ACTIVITY 1-like
**24.**	A0A6P6TCJ6	Peroxidase
**25.**	A0A6P6TDH7	Ras-related protein RHN1
**26.**	A0A6P6TFG1	Ras-related protein Rab11D-like
**27.**	A0A6P6TID6	Rac-like GTP-binding protein RHO1
**28.**	A0A6P6TQI7	Probable serine/threonine-protein kinase PBL19
**29.**	A0A6P6TRF1	Serine/threonine-protein kinase BSK7-like
**30.**	A0A6P6TS63	Binding partner of ACD11 1-like
**31.**	A0A6P6TSS3	LOW QUALITY PROTEIN: SNARE-interacting protein KEULE-like
**32.**	A0A6P6TU70	GTP-binding protein YPTM2-like isoform X2
**33.**	A0A6P6TU71	Profilin
**34.**	A0A6P6TW37	CASP-like protein
**35.**	A0A6P6U0Y2	GABA transporter 1-like
**36.**	A0A6P6U1R1	Profilin
**37.**	A0A6P6U2C4	Nucleobase-ascorbate transporter 12-like
**38.**	A0A6P6U6D2	Protein IN2-1 homolog B-like isoform X1
**39.**	A0A6P6U7A8	Polyol transporter 1
**40.**	A0A6P6U888	Serine/threonine-protein kinase BSK1-like
**41.**	A0A6P6U9L0	Plasma membrane-associated cation-binding protein 1-like
**42.**	A0A6P6UE29	Probable serine/threonine-protein kinase PBL9 isoform X1

**43.**	A0A6P6UEA3	Serine/threonine-protein kinase BSK5-like
**44.**	A0A6P6UFS0	Probable serine/threonine-protein kinase PBL7 isoform X1
**45.**	A0A6P6UFU7	Copper transport protein
**46.**	A0A6P6UG28	Plasma membrane-associated cation-binding protein 1-like
**47.**	A0A6P6UI44	Phosphoinositide phospholipase C
**48.**	A0A6P6UJY3	Uncharacterized protein LOC113712075
**49.**	A0A6P6UM98	Nucleobase-ascorbate transporter 3-like isoform X1
**50.**	A0A6P6UMD3	Ras-related protein RABB1b
**51.**	A0A6P6UNF4	LOW QUALITY PROTEIN: phosphoprotein ECPP44-like
**52.**	A0A6P6UPC1	Patellin-3-like
**53.**	A0A6P6UQT9	Ras-related protein RABA5b-like
**54.**	A0A6P6UR00	Membrane-anchored ubiquitin-fold protein
**55.**	A0A6P6USD0	Oligopeptide transporter 4-like
**56.**	A0A6P6UU98	Nucleobase-ascorbate transporter 3-like
**57.**	A0A6P6UVC2	Glycolipid transfer protein 1
**58.**	A0A6P6UXE9	Ras-related protein RABC1
**59.**	A0A6P6UY40	Non-specific serine/threonine protein kinase
**60.**	A0A6P6V4E2	UPF0664 stress-induced protein C29B12.11c-like isoform X1
**61.**	A0A6P6VCU2	Serine/threonine-protein kinase PBS1-like
**62.**	A0A6P6VD95	Temperature-induced lipocalin-1-like isoform X2
**63.**	A0A6P6VFZ0	CASP-like protein
**64.**	A0A6P6VNZ7	Elicitor-responsive protein 1
**65.**	A0A6P6VQQ6	GABA transporter 1-like
**66.**	A0A6P6VRH0	Ras-related protein RABE1c-like
**67.**	A0A6P6VRQ0	Secretory carrier-associated membrane protein
**68.**	A0A6P6VXE8	Ras-related protein RABD2a-like
**69.**	A0A6P6VXK5	Ras-related protein RABD2a
**70.**	A0A6P6VY15	Serine/threonine-protein kinase BSK2-like
**71.**	A0A6P6VZW3	Probable serine/threonine-protein kinase PBL7
**72.**	A0A6P6W193	Uncharacterized protein LOC113727819
**73.**	A0A6P6W208	Ras-related protein RABE1c isoform X1
**74.**	A0A6P6W3F2	Metal-nicotianamine transporter YSL2-like
**75.**	A0A6P6W6S9	Receptor-like protein kinase HERK 1
**76.**	A0A6P6WAQ1	Vesicle-associated protein 4-2-like
**77.**	A0A6P6WB36	Non-classical arabinogalactan protein 31-like
**78.**	A0A6P6WDA6	Ras-related protein RABA2a
**79.**	A0A6P6WDA8	Rac-like GTP-binding protein 5
**80.**	A0A6P6WDQ1	Oligopeptide transporter 4-like
**81.**	A0A6P6WE80	Guanine nucleotide-binding protein subunit beta-2 isoform X1
**82.**	A0A6P6WEP6	Cinnamoyl-CoA reductase 2-like isoform X1
**83.**	A0A6P6WH53	Uncharacterized protein LOC113731820 isoform X1
**84.**	A0A6P6WHH8	Uncharacterized protein LOC113732991
**85.**	A0A6P6WHI7	Ras-related protein RABA5b-like
**86.**	A0A6P6WIF6	Probable serine/threonine-protein kinase At1g01540

**87.**	A0A6P6WMF9	Novel plant SNARE 13-like
**88.**	A0A6P6WXE8	Berberine bridge enzyme-like 18
**89.**	A0A6P6X034	Protein SRC2-like
**90.**	A0A6P6X220	MAPK kinase substrate protein At1g80180-like
**91.**	A0A6P6X616	LOW QUALITY PROTEIN: PTI1-like tyrosine-protein kinase
**92.**	A0A6P6X7W9	Plasma membrane ATPase
**93.**	A0A6P6X8Z7	Protein NRT1/ PTR FAMILY 2.11-like
**94.**	A0A6P6X901	Calcium-dependent protein kinase 10-like
**95.**	A0A6P6XDH2	Ras-related protein Rab7
**96.**	A0A6P6XE46	PTI1-like tyrosine-protein kinase At3g15890
**97.**	A0A6P6XEJ1	Protein-serine/threonine phosphatase
**98.**	A0A6P6XH31	Protein ASPARTIC PROTEASE IN GUARD CELL 1-like isoform X1
**99.**	A0A6P6XH53	Protein PLANT CADMIUM RESISTANCE 8-like
**100.**	S4NPP0	Aquaporin PIP1-3-like

Table S5: List of the 93 proteins exclusive to the 100k×*g* fraction, clustered as Plasma membrane (GO:0005886).

	**UniProt**	**Protein identification**
**1.**	A0A6P6S9C1	Protein NOI4-like
**2.**	A0A6P6S9G2	Protein NOI4-like
**3.**	A0A6P6SA02	Protein NDR1-like
**4.**	A0A6P6SHH5	FT-interacting protein 7-like
**5.**	A0A6P6SIP3	Serine/threonine-protein kinase isoform X1
**6.**	A0A6P6SK98	Calcium-dependent protein kinase 8-like
**7.**	A0A6P6SQQ6	Vacuolar protein sorting-associated protein 32 homolog 2
**8.**	A0A6P6SR90	Auxin transporter-like protein 2
**9.**	A0A6P6SRR2	Probable serine/threonine-protein kinase PBL17
**10.**	A0A6P6SRV0	Sugar transport protein 7-like
**11.**	A0A6P6SUL9	FT-interacting protein 7-like
**12.**	A0A6P6SVT4	Calcium-dependent protein kinase 8-like
**13.**	A0A6P6SYV1	Heavy metal-associated isoprenylated plant protein 6
**14.**	A0A6P6SZX5	Probable serine/threonine-protein kinase PBL8 isoform X1
**15.**	A0A6P6T2R3	VAMP-like protein YKT61
**16.**	A0A6P6T3C0	Auxin transporter-like protein 2
**17.**	A0A6P6T443	Temperature-induced lipocalin-1-like
**18.**	A0A6P6T638	Probable receptor-like protein kinase At2g23200
**19.**	A0A6P6T779	Nucleobase-ascorbate transporter 11-like
**20.**	A0A6P6T9D6	Uncharacterized protein LOC113698779
**21.**	A0A6P6T9G7	non-specific serine/threonine protein kinase
**22.**	A0A6P6TA99	Protein MID1-COMPLEMENTING ACTIVITY 1-like
**23.**	A0A6P6TDH7	Ras-related protein RHN1
**24.**	A0A6P6TFG1	Ras-related protein Rab11D-like
**25.**	A0A6P6TID6	Rac-like GTP-binding protein RHO1
**26.**	A0A6P6TQI7	Probable serine/threonine-protein kinase PBL19
**27.**	A0A6P6TRF1	Serine/threonine-protein kinase BSK7-like
**28.**	A0A6P6TS63	Binding partner of ACD11 1-like
**29.**	A0A6P6TSS3	LOW QUALITY PROTEIN: SNARE-interacting protein KEULE-like
**30.**	A0A6P6TU70	GTP-binding protein YPTM2-like isoform X2
**31.**	A0A6P6TW37	CASP-like protein
**32.**	A0A6P6U0Y2	GABA transporter 1-like
**33.**	A0A6P6U2C4	Nucleobase-ascorbate transporter 12-like
**34.**	A0A6P6U6D2	Protein IN2-1 homolog B-like isoform X1
**35.**	A0A6P6U7A8	Polyol transporter 1
**36.**	A0A6P6U888	Serine/threonine-protein kinase BSK1-like
**37.**	A0A6P6U9L0	Plasma membrane-associated cation-binding protein 1-like
**38.**	A0A6P6UE29	Probable serine/threonine-protein kinase PBL9 isoform X1
**39.**	A0A6P6UEA3	Serine/threonine-protein kinase BSK5-like
**40.**	A0A6P6UFS0	Probable serine/threonine-protein kinase PBL7 isoform X1
**41.**	A0A6P6UFU7	Copper transport protein
**42.**	A0A6P6UG28	Plasma membrane-associated cation-binding protein 1-like

**43.**	A0A6P6UI44	Phosphoinositide phospholipase C
**44.**	A0A6P6UJY3	Uncharacterized protein LOC113712075
**45.**	A0A6P6UM98	Nucleobase-ascorbate transporter 3-like isoform X1
**46.**	A0A6P6UMD3	Ras-related protein RABB1b
**47.**	A0A6P6UNF4	LOW QUALITY PROTEIN: phosphoprotein ECPP44-like
**48.**	A0A6P6UPC1	Patellin-3-like
**49.**	A0A6P6UQT9	Ras-related protein RABA5b-like
**50.**	A0A6P6UR00	Membrane-anchored ubiquitin-fold protein
**51.**	A0A6P6USD0	Oligopeptide transporter 4-like
**52.**	A0A6P6UU98	Nucleobase-ascorbate transporter 3-like
**53.**	A0A6P6UVC2	Glycolipid transfer protein 1
**54.**	A0A6P6UXE9	Ras-related protein RABC1
**55.**	A0A6P6UY40	non-specific serine/threonine protein kinase
**56.**	A0A6P6V4E2	UPF0664 stress-induced protein C29B12.11c-like isoform X1
**57.**	A0A6P6VCU2	Serine/threonine-protein kinase PBS1-like
**58.**	A0A6P6VD95	Temperature-induced lipocalin-1-like isoform X2
**59.**	A0A6P6VFZ0	CASP-like protein
**60.**	A0A6P6VNZ7	Elicitor-responsive protein 1
**61.**	A0A6P6VQQ6	GABA transporter 1-like
**62.**	A0A6P6VRH0	Ras-related protein RABE1c-like
**63.**	A0A6P6VRQ0	Secretory carrier-associated membrane protein
**64.**	A0A6P6VXE8	Ras-related protein RABD2a-like
**65.**	A0A6P6VXK5	Ras-related protein RABD2a
**66.**	A0A6P6VY15	Serine/threonine-protein kinase BSK2-like
**67.**	A0A6P6VZW3	Probable serine/threonine-protein kinase PBL7
**68.**	A0A6P6W193	Uncharacterized protein LOC113727819
**69.**	A0A6P6W208	Ras-related protein RABE1c isoform X1
**70.**	A0A6P6W3F2	Metal-nicotianamine transporter YSL2-like
**71.**	A0A6P6W6S9	Receptor-like protein kinase HERK 1
**72.**	A0A6P6WAQ1	Vesicle-associated protein 4-2-like
**73.**	A0A6P6WDA6	Ras-related protein RABA2a
**74.**	A0A6P6WDA8	Rac-like GTP-binding protein 5
**75.**	A0A6P6WDQ1	Oligopeptide transporter 4-like
**76.**	A0A6P6WE80	Guanine nucleotide-binding protein subunit beta-2 isoform X1
**77.**	A0A6P6WEP6	Cinnamoyl-CoA reductase 2-like isoform X1
**78.**	A0A6P6WH53	Uncharacterized protein LOC113731820 isoform X1
**79.**	A0A6P6WHH8	Uncharacterized protein LOC113732991
**80.**	A0A6P6WHI7	Ras-related protein RABA5b-like
**81.**	A0A6P6WIF6	Probable serine/threonine-protein kinase At1g01540
**82.**	A0A6P6WMF9	Novel plant SNARE 13-like
**83.**	A0A6P6X034	Protein SRC2-like
**84.**	A0A6P6X220	MAPK kinase substrate protein At1g80180-like
**85.**	A0A6P6X616	LOW QUALITY PROTEIN: PTI1-like tyrosine-protein kinase At3g15890
**86.**	A0A6P6X7W9	Plasma membrane ATPase

**87.**	A0A6P6X8Z7	Protein NRT1/ PTR FAMILY 2.11-like
**88.**	A0A6P6X901	Calcium-dependent protein kinase 10-like
**89.**	A0A6P6XDH2	Ras-related protein Rab7
**90.**	A0A6P6XE46	PTI1-like tyrosine-protein kinase At3g15890
**91.**	A0A6P6XEJ1	protein-serine/threonine phosphatase
**92.**	A0A6P6XH53	Protein PLANT CADMIUM RESISTANCE 8-like
**93.**	S4NPP0	Aquaporin PIP1-3-like

Table S6: GO analysis and list of proteins exclusive to the 125k×*g* fraction.

	Cellular Component (Gene Ontology)				
**GO-term**	**Description**	**Count in network**	**Strength**	**Signal**	**False Discovery Rate**
GO:0005576	Extracellular region	30 of 2491	0.96	2.00	$1.84e^{-18}$
GO:0030312	External encapsulating structure	8 of 366	1.21	1.22	$1.73e^{-05}$
GO:0005618	Cell wall	7 of 330	1.20	1.07	$7.84e^{-05}$
GO:0048046	Apoplast	9 of 825	0.91	0.81	0.00030
GO:0009505	Plant type cell wall	5 of 261	1.16	0.67	0.0044

Table S7: List of the 30 proteins exclusive to 125k×*g* fraction, clustered in Extracellular region (GO:0005576).

	UniProt	Protein identification
**1.**	A0A6P6S5G0	Xyloglucan endotransglucosylase/hydrolase
**2.**	A0A6P6S7H5	Purple acid phosphatase
**3.**	A0A6P6SDY0	Chitinase
**4.**	A0A6P6SFE6	Pectinesterase
**5.**	A0A6P6SHP0	Peroxidase
**6.**	A0A6P6T1A6	Non-reducing end alpha-L-arabinofuranosidase
**7.**	A0A6P6T573	L-ascorbate oxidase
**8.**	A0A6P6TAK2	Beta-galactosidase
**9.**	A0A6P6TKM2	Non-specific serine/threonine protein kinase
**10.**	A0A6P6TZ47	Glucan endo-1,3-beta-glucosidase 14
**11.**	A0A6P6U0C2	Serine carboxypeptidase-like 7 isoform X1
**12.**	A0A6P6U1T4	Beta-fructofuranosidase, insoluble isoenzyme CWINV1-like
**13.**	A0A6P6U2J7	Carboxypeptidase
**14.**	A0A6P6U716	Berberine bridge enzyme-like 8
**15.**	A0A6P6U751	Berberine bridge enzyme-like 21
**16.**	A0A6P6UGU5	Beta-galactosidase
**17.**	A0A6P6UH03	Serine carboxypeptidase-like 11 isoform X1
**18.**	A0A6P6UPP0	Carboxypeptidase
**19.**	A0A6P6UWG7	Serine carboxypeptidase-like 7 isoform X1
**20.**	A0A6P6UX35	Berberine bridge enzyme-like 8 isoform X1
**21.**	A0A6P6VKR8	Chitinase
**22.**	A0A6P6VQW7	Xyloglucan endotransglucosylase/hydrolase
**23.**	A0A6P6VR56	Low-temperature-induced cysteine proteinase-like
**24.**	A0A6P6VXL3	Pectinesterase
**25.**	A0A6P6WB63	Pathogenesis-related protein PR-1 type-like
**26.**	A0A6P6WD89	Alpha-L-fucosidase 2-like
**27.**	A0A6P6WGW2	Pectinesterase
**28.**	A0A6P6WHK0	Rapid alkalinization factor-like
**29.**	A0A6P6WRJ0	Berberine bridge enzyme-like 18
**30.**	A0A6P6XD04	Alpha-galactosidase

Table S8: List of plant EV marker protein accessions in the subset of shared and exclusive proteins from either the 100k×*g* or 125k×*g* fractions. Markers are listed by accession number and organized into five functional categories. N.d., not detected. Gene IDs in brackets represent synonyms of the preceding entry, according to UniprotKB database. Shared accessions and gene loci are highlighted in bold. The symbol ✓ indicates the presence of shared proteins in either the 100k×*g* or 125k×*g* fraction.

**Class**	**Gene ID (Coffea arabica) (570 total accessions)**	**Shared accessions (232 accessions)**	**Accessions exclusive to the 100k×*g* fraction (267 accessions)**	**Accessions exclusive to the 125k×*g* fraction (71 accessions)**
**Group 1: transmembrane or GPI-anchored proteins**				
**TET8**	Tetraspanin-8-like			
Tetraspanins membrane proteins [23,33-36]	**LOC113696066**	**A0A6P6SZS3**	✓	✓
	**(LOC113692822)**			
	**(LOC113692860)**			
	Tetraspanin-3-like			
	**LOC113708417**	**A0A6P6U8X8**	✓	✓
**PEN1**	Syntaxin 121-like			
Penetration1 intraluminal protein Syntaxins [34,36]	**LOC113691528**	**A0A6P6SI59**	✓	✓
	**(LOC113691404)**			
	**LOC113693839**	**A0A6P6SR51**	✓	✓
	Syntaxin 132-like			
	**LOC113688935**	**A0A6P6S8V0**	✓	✓
	Syntaxin 71-like			
	LOC113695867	N.d.	A0A6P6SYF6	N.d.
	SNARE proteins			
	LOC113733827	N.d.	A0A6P6WMF9	N.d.
**Ser/Thr-protein kinases**	Non-specific Ser/Thr protein kinases			

Classified together with G-type lectin S-receptor-like and involved in stress [9,12]	**LOC113692486**	**A0A6P6SLK0**	✓	✓
	**LOC113728539**	**A0A6P6W3N9**	✓	✓
	**LOC113719237**	**A0A6P6VB63**	✓	✓
	**LOC113731548**	**A0A6P6WCB7**	✓	✓
	**LOC113712339**	**A0A6P6UP73**	✓	✓
	LOC113710377	N.d.	A0A6P6UEA3	N.d.
	LOC113708644	N.d.	A0A6P6U888	N.d.
	LOC113727003	N.d.	A0A6P6VY15	N.d.
	LOC113709797	N.d.	A0A6P6UE29	N.d.
	LOC113734531	N.d.	A0A6P6WNS2	N.d.
	LOC113710522	N.d.	A0A6P6UFS0	N.d.
	LOC113719010	N.d.	A0A6P6VCU2	N.d.
	LOC113693916	N.d.	A0A6P6SRR2	N.d.
	LOC113696095	N.d.	A0A6P6SZX5	N.d.
	LOC113711145	N.d.	A0A6P6UK42	N.d.
	LOC113691006	N.d.	A0A6P6SGK4	N.d.
	LOC113733022	N.d.	A0A6P6WHM9	N.d.
	LOC113734857	N.d.	A0A6P6WQ76	N.d.
	LOC113733220	N.d.	A0A6P6WIF6	N.d.
	LOC113719120	N.d.	A0A6P6VAM9	N.d.
	LOC113743231	N.d.	N.d.	A0A6P6XIE3
	LOC113701471	N.d.	N.d.	A0A6P6TIS5
	LOC113701906	N.d.	N.d	A0A6P6TKM2
	Probable Ser/Thr-protein kinase PBL19 LOC113703530	N.d.	A0A6P6TQI7	N.d.
	Cysteine-rich receptor-like protein kinase 15 isoform X1 LOC113739755	N.d.	A0A6P6X5E1	N.d.

	Cold-responsive protein kinase 1-like			
	LOC113743402	N.d.	A0A6P6XMC1	N.d.
	LOC113687865	N.d.	A0A6P6S4V8	N.d.
	Probable Ser/Thr-protein kinase PBL7			
	LOC113728414	N.d.	A0A6P6VZW3	N.d.
	LRR receptor-like Ser/Thr-protein kinases			
	LOC113708676	N.d.	N.d.	A0A6P6U911
	LOC113743479	N.d.	N.d.	A0A6P6XHV5
	LOC113696248	N.d.	N.d.	A0A6P6T071
	Receptor-like Ser/Thr-protein kinases			
	**LOC113717205**	**A0A6P6V724**	✓	✓
	LOC113719566	N.d.	A0A6P6VD97	N.d.
	LOC113706296	N.d.	N.d.	A0A6P6U0G6
	LOC113719651	N.d.	N.d.	A0A6P6VFH6
**Group 2: cytosolic proteins recovered in EVs**				
**HSP70**<br>Heat shock protein 70 [12]	Heat shock cognate 70 kDa protein-like			
	LOC113708791	N.d.	N.d.	A0A6P6U8W3
**ANXA**	Annexin			
Annexin [12,23,37]	LOC113713327	N.d.	A0A6P6UPT4	N.d.
**PATL3**	Patellin 3			
Patellin or Patellin-like intraluminal proteins [34,36-38]	LOC113712940	N.d.	A0A6P6UPC1	N.d.

**Actins**	Actins & Actin-like proteins			
Actins & Actin-like proteins				
[35,39]	**LOC113725474**	**A0A6P6VPI2**	✓	✓
	**(LOC113730480)**			
	**(LOC113730498)**			
	LOC113701041	N.d.	A0A6P6TGY8	N.d.
	(LOC113698016)			
	LOC113695736	N.d.	A0A6P6SXD5	N.d.
	LOC113711906	N.d.	N.d.	A0A6P6UK92
**GTPase Rab-type**	Rac-like GTP-binding protein 5			
Rab-like GTPase marker of MVB (multivesicular body)				
[5,23,33]	**LOC113726249**	**A0A6P6VRK8**	✓	✓
	LOC113701083	N.d.	A0A6P6TID6	N.d.
	LOC113732028	N.d.	A0A6P6WDA8	N.d.
	Ras-related protein RABD1			
	**LOC113731936**	**A0A6P6WFC6**	✓	✓
	Ras-related protein			
	RABD2a-like	N.d.	A0A6P6VXE8	N.d.
	LOC113727338	N.d.	A0A6P6VXK5	N.d.
	LOC113726882			
	Ras-related protein			
	RABE1c-like	**A0A6P6T3A0**	✓	✓
	**LOC113697127**	N.d.	A0A6P6UXE9	N.d.
	LOC113714582	N.d.	A0A6P6W208	N.d.
	LOC113728824	N.d.	A0A6P6VRH0	N.d.
	LOC113725513			
	Ras-related protein Rab11C			
	**LOC113710301**	**A0A6P6UEL8**	✓	✓

Ras-related protein Rab11D-like			
LOC113700090	N.d.	A0A6P6TFG1	N.d.
Ras-related protein Rab7			
LOC113742257	N.d.	A0A6P6XDH2	N.d.
GTP-binding protein YPTM2-like			
LOC113704232	N.d.	A0A6P6TU70	N.d.
LOC113704232	N.d.	N.d.	A0A6P6TTR0
GTP-binding nuclear protein			
LOC113693625	N.d.	A0A6P6SQ71	N.d.
LOC113703624	N.d.	A0A6P6TRT9	N.d.
Ras-related protein RABA1			
LOC113689020	N.d.	A0A6P6S926	N.d.
LOC113726201	N.d.	A0A6P6VUK9	N.d.
LOC113726201	N.d.	N.d.	A0A6P6VUK9
Ras-related protein RABA2a			
LOC113731935	N.d.	A0A6P6WDA6	N.d.
Ras-related protein RABA5b-like			
LOC113732427	N.d.	A0A6P6WHI7	N.d.
LOC113713227	N.d.	A0A6P6UQT9	N.d.
Ras-related protein RABB1			
LOC113712424	N.d.	A0A6P6UMD3	N.d.

	LOC113690727	N.d.	A0A6P6SFE4	N.d.
	Ras-related protein RABH1b-like			
	LOC113726914	N.d.	A0A6P6VXP5	N.d.
	LOC113732054	N.d.	A0A6P6WI73	N.d.
	Ras-related protein RABF2b			
	LOC113709103	N.d.	A0A6P6UB04	N.d.
	Ras-related protein RHN1			
	LOC113700343	N.d.	A0A6P6TDH7	N.d.
**RIN4**	RIN4/NOI4			
RPM1-Interacting Protein 4 [5,37,40]	LOC113688795	N.d.	A0A6P6S9G2	N.d.
	LOC113688752 (LOC113743193)	N.d.	A0A6P6S9C1	N.d.
**ESCRT-related proteins**	ESCRT-related protein CHMP1B			
Involved in MVB (multivesicular body) and ILVs formation (intraluminal vesicles) Endosomal Sorting Complex Required for Transport involves about 20 proteins (e.g. ESCRT-0, -I, -II, and -III, AAA ATPase Vps4 complex) [5,41]	LOC113742273	N.d.	A0A6P6XDK0	N.d.
	Vacuolar protein sorting-associated protein 2 homolog 1			
	LOC113693884	N.d.	A0A6P6SRQ0	N.d.
	Vacuolar protein sorting-associated protein 24 homolog 1-like			
	LOC113742231	N.d.	A0A6P6XDI5	N.d.

	Vacuolar protein sorting-associated protein 32 homolog 2			
	LOC113693726	N.d.	A0A6P6SQQ6	N.d.
	Vacuolar protein sorting-associated protein 20 homolog 2			
	LOC113735206	N.d.	A0A6P6WTH6	N.d.
**Ser carboxypeptidase**	Ser carboxypep-like 7			
Serine carboxypeptidases could act in plant as acyl transferase [35]	**LOC113714935**	**A0A6P6UWG9**	✓	✓
	**LOC113704913**	**A0A6P6TW52**	✓	✓
	Ser carboxypep-like 11			
	LOC113714934	N.d.	N.d.	A0A6P6UWG7
	LOC113705873	N.d.	N.d.	A0A6P6U0C2
	LOC113710936	N.d.	N.d.	A0A6P6UH03
**Group 3: major components of non-EV co-isolated structures**				
**None**				
**Group 4: transmembrane, lipid bound and soluble proteins associated to other intracellular compartments than PM/endosomes**				
**Mitochondrion**	**LOC113688097**	**A0A6P6S800**	✓	✓
	LOC113703554	N.d.	A0A6P6TT16	N.d.
	LOC113728131	N.d.	A0A6P6W0K2	N.d.
**Plastid**	**LOC113703862**	**A0A6P6TU06**	✓	✓
	**LOC113742299**	**A0A6P6XIQ4**	✓	✓
	LOC113695961	N.d.	A0A6P6SYV2	N.d.
	LOC113742593	N.d.	A0A6P6XH31	N.d.
	LOC113690500	N.d.	A0A6P6SEJ9	N.d.

	LOC113714316	N.d.	A0A6P6UTE2	N.d.
	LOC113693856	N.d.	A0A6P6SQZ2	N.d.
	LOC113730040	N.d.	A0A6P6W843	N.d.
	LOC113720169	N.d.	A0A6P6VD95	N.d.
	LOC113697378	N.d.	A0A6P6T443	N.d.
	LOC113709314	N.d.	A0A6P6UC84	N.d.
	LOC113687987	N.d.	A0A6P6S5D4	N.d.
	LOC113691812	N.d.	A0A6P6SJA2	N.d.
	LOC113732299	N.d.	A0A6P6WEP6	N.d.
	LOC113690490	N.d.	N.d.	A0A6P6SDI9
	LOC113688268	N.d.	N.d.	A0A6P6S7H5
	LOC113723998	N.d.	N.d.	A0A6P6VIF7
**Nucleus**	**LOC113689178**	**A0A6P6SAM3**	✓	✓
	**LOC113726310**	**A0A6P6VRR9**	✓	✓
	**LOC113736196**	**A0A6P6WTZ7**	✓	✓
	LOC113714295	N.d.	A0A6P6UTT1	N.d.
	LOC113702812	N.d.	A0A6P6TNY8	N.d.
	LOC113714732	N.d.	A0A6P6UV84	N.d.
	LOC113701787	N.d.	A0A6P6TLF0	N.d.
	LOC113740276	N.d.	A0A6P6X9T8	N.d.
	LOC113725863	N.d.	A0A6P6VQZ2	N.d.
	LOC113705230	N.d.	A0A6P6TWU4	N.d.
	LOC113703582	N.d.	N.d.	A0A6P6TT42
	LOC113715842	N.d.	N.d.	A0A6P6UZS0
	LOC113730577	N.d.	N.d.	A0A6P6W732
**Peroxisomes**	LOC113717581	N.d.	A0A6P6V545	N.d.
	LOC113706930	N.d.	A0A6P6U2T6	N.d.
	LOC113731485	N.d.	N.d.	A0A6P6WD89
**Group 5: Cell Wall Remodelling/Degrading Enzymes (CWREs/CWDEs) and pathogenesis related (PR) proteins**				
**Glucosidases**	glucan endo-1,3-beta-D-glucosidase			

Glycosyl hydrolase family 3 proteins (α or β-1,3-Glucosidase, generally carbohydrate hydrolitic enzymes) [12,23,35,14]	**LOC113731292**	**A0A6P6W9Z2**	✓	✓
	**LOC113714387**	**A0A6P6UTB2**	✓	✓
	**LOC113712345**	**A0A6P6UPB4**	✓	✓
	**LOC113697105**	**A0A6P6T231**	✓	✓
	**LOC113694078**	**A0A6P6SRV9**	✓	✓
	**LOC113712534**	**A0A6P6ULW7**	✓	✓
	**LOC113692581**	**A0A6P6SLP2**	✓	✓
	**LOC113699506**	**A0A6P6TB57**	✓	✓
	**LOC113710273**	**A0A6P6UEU9**	✓	✓
	**LOC113694127**	**A0A6P6SSP8**	✓	✓
	**LOC113694866**	**A0A6P6SUV4**	✓	✓
	**LOC113696215**	**A0A6P6T0G8**	✓	✓
	**LOC113692647**	**A0A6P6SM05**	✓	✓
	LOC113705815	N.d.	N.d.	A0A6P6TZ47
	LOC113688069	N.d.	N.d.	A0A6P6S5V4
	α or β-galactosidase			
	**LOC113708085**	**A0A6P6U6L5**	✓	✓
	**LOC113743043**	**A0A6P6XIZ3**	✓	✓
	**LOC113692453**	**A0A6P6SL89**	✓	✓
	**LOC113742826**	**A0A6P6XKP1**	✓	✓
	LOC113740457	N.d.	N.d.	A0A6P6XD04
	LOC113699224	N.d.	N.d.	A0A6P6TAK2
	LOC113710894	N.d.	N.d.	A0A6P6UGU5
**Pectinesterases**	Pectinesterase			
Cell wall biogenesis/degradation Pectinesterase/pectinesterase inhibitor 51 [12,35,37]	**LOC113730832**	**A0A6P6W961**	✓	✓
	**LOC113727733**	**A0A6P6VXM4**	✓	✓
	**LOC113692644**	**A0A6P6SM06**	✓	✓
	**LOC113724886**	**A0A6P6VLH8**	✓	✓
	**LOC113729938**	**A0A6P6W7I9**	✓	✓
	LOC113690938	N.d.	N.d.	A0A6P6SFE6
	LOC113732901	N.d.	N.d.	A0A6P6WGW2
	LOC113727732	N.d.	N.d.	A0A6P6VXL3

**Chitinases**<br>Class V chitinases and endochitinases [9,35]	Chitinases & endochitinases			
	**LOC113709552**	**A0A6P6UCD9**	✓	✓
	**LOC113693790**	**A0A6P6SR39**	✓	✓
	**LOC113728487**	**A0A6P6W2B9**	✓	✓
	**LOC113695870**	**A0A6P6SYG0**	✓	✓
	**LOC113692994**	**A0A6P6SNE2**	✓	✓
	**LOC113716360**	**A0A6P6V0U3**	✓	✓
	**LOC113702310**	**A0A6P6TL51**	✓	✓
	**LOC113692387**	**A0A6P6SKC8**	✓	✓
	**LOC113719150**	**A0A6P6VBR3**	✓	✓
	**LOC113736853**	**A0A6P6WWQ5**	✓	✓
	**LOC113735013**	**A0A6P6WQJ8**	✓	✓
	**LOC113736851**	**A0A6P6X1X9**	✓	✓
	LOC113729974	N.d.	A0A6P6W7R0	N.d.
	LOC113735015	N.d.	N.d.	A0A6P6WSL0
	LOC113736861	N.d.	N.d.	A0A6P6X1Y7
	LOC113724934	N.d.	N.d.	A0A6P6VKR8
	LOC113690342	N.d.	N.d.	A0A6P6SDY0
**Peroxidases**	Peroxidases			
[35,42]	**LOC113691808**	**A0A6P6SJ83**	✓	✓
	**LOC113694958**	**A0A6P6SV85**	✓	✓
	**LOC113730275**	**A0A6P6W9A2**	✓	✓
	**LOC113743511**	**A0A6P6XI18**	✓	✓
	**LOC113701179**	**A0A6P6THJ8**	✓	✓
	**LOC113697863**	**A0A6P6T4J5**	✓	✓
	**LOC113690410**	**A0A6P6SE81**	✓	✓
	LOC113726664	N.d.	A0A6P6VTC4	N.d.
	LOC113699344	N.d.	A0A6P6TCJ6	N.d.
	LOC113691066	N.d.	N.d.	A0A6P6SHP0
	LOC113726664	N.d.	N.d.	A0A6P6VTC4

**BBE-like proteins**<br>Berberine bridge enzyme-like 8, -18 and -21, related to oxidative reactions [12]	Berberine bridge enzyme-like 21			
	**LOC113710735**	**A0A6P6UHF7**	✓	✓
	**LOC113707773**	**A0A6P6U6X2**	✓	✓
	Berberine bridge enzyme-like 18			
	**LOC113707934**	**A0A6P6U6Q2**	✓	✓
	LOC113737058	N.d.	A0A6P6WXE8	N.d.
	LOC113707711	N.d.	N.d.	A0A6P6U751
	LOC113735165	N.d.	N.d.	A0A6P6WRJ0
	LOC113715336	N.d.	N.d.	A0A6P6UX35
	Berberine bridge enzyme-like 8			
	LOC113707817	N.d.	N.d	A0A6P6U716
